# Evaluation of the quality and safety of commercial complementary foods: Implications for nutrient adequacy and conformance with national and international standards

**DOI:** 10.1371/journal.pone.0294068

**Published:** 2024-02-21

**Authors:** Elroe Mario, Abera Belay, Endale Amare

**Affiliations:** 1 Department of Food Science and Applied Nutrition, and Bioprocessing and Biotechnology Center of Excellence, Addis Ababa Science and Technology University, Addis Ababa, Ethiopia; 2 Food Science and Nutrition Research Directorate, Ethiopian Public Health Institute, Addis Ababa, Ethiopia; International Institute of Tropical Agriculture (IITA), ZAMBIA

## Abstract

Optimal nutrition in early childhood increases growth and development while preventing morbidity and mortality in later life. This study focused on the quality and safety of 32 commercially produced complementary foods collected from supermarkets in Addis Ababa, Ethiopia. The proximate composition (moisture, protein, fat, crude fibre, and ash); and the mineral profile (calcium, iron, zinc, manganese, and magnesium) were determined using the AOAC method. The determination of antinutritional factors (Condensed tannin and oxalate) was done using a UV spectrophotometer. A rapid visco analyzer was used to measure the rheological property. The microbial load of commercially produced complimentary foods was identified using aerobic colony counts for mold and yeast. Labeling practice was evaluated using the WHO and Ethiopian standard tools. The results of proximate compositions were: protein (0.92–18.16 g/100g), fat (0.63–6.44 g/100g), crude fiber (1.04–13.2 g/100g), energy (410–337 kcal/100g), moisture (0.03–17 g/100g), and ash (**0.60–4.67 g/100g**). The protein and fat content of all the products is below international standards. Only three products met the standards for energy. Moisture and ash contents partially met the requirement, while all of the carbohydrate contents of the products fell under the specified standard. The lowest and highest mineral contents of the products were: Fe (1.38 to 15.10 mg/100g), Zn (0.64 to 6.78 mg/100g), Ca (30.55 to 364.45 mg/100g), Mg (1.2 to 34.2 mg/100g), and Mn (0.80 to 32 mg/100g). Based on these, 21.5% of the foods met the Fe standard, and 31.5% didn’t meet the Zn standard. The Ca and Mg of all the products met the requirement. Except for one product, all met standards. The highest and lowest results for the tannin and oxalate content of the products were 49.20 to 90.09 mg/100g and 0.47 to 30.10 mg/100g, respectively and this shows that the products are below the permissible range for tannin and oxalate. The counts of yeast and mold were 0.00–2.95 log_10_ cfu/g and 0.00–2.91 log_10_ cfu/g, respectively. **Only one product fell below the standard for yeast count**, and none of the products showed a mold count that exceeded the standard. The final viscosity was 63.5–3476 RVU, and only 31.25% of the samples fell under the permissible peak viscosity range, of 83–250 RVU. Thus, regular monitoring of the raw material and processing trends and the inclusion of animal sources in the raw material are suggested for having well-enriched complementary foods. Regulatory bodies should also conduct frequent market surveillance to safeguard the health of the consumer.

## Introduction

Nutrition in the first 1000 days of life is critical for growth and development [1]. These 1000 days encompass complementary feeding, a bridging period from six to twenty-four months of age when children learn to increase family food intake via the gradual introduction of solids and liquids alongside their usual breast milk [2]. Breastfeeding is the gold standard for infant feeding, and it is encouraged for two years and beyond. However, at around 6 months of age, an infant’s requirements for energy, protein, and other nutrients (particularly iron and zinc) cannot be met by breast milk alone [3]. In addition, requirements for many micronutrients (specifically, vitamins B_**1**_, B_**2**_, B_**3**_, B_**6**_, B_**12**,_ and calcium) increase from the first to the second year. In addition to that, infants reach physiological (chewing, swallowing, digestion, and excretion) and neurological stage of development that enables them to be fed other foods rather than breast milk [4].

The introduction of complementary foods is a ‘window of opportunity’ for children. This window may be considered in terms of specific nutritional requirements at the time, as a developmental opportunity in terms of receptiveness to varied textures of foods, or may be considered the optimal timing of exposure to food allergens to foster the development of immune tolerance [5]. During this stage, the nutritional quality of the food in the child’s diet becomes even more important [6]. In addition to meeting specific nutritional requirements, this time comes at a delicate developmental stage as the child begins to focus on the development of gross motor skills and begins to assert independence [3].

The foods chosen for children by mothers/caretakers are dependent on complex social, economic, cultural, and political determinants of the food environment. Since their ‘invention’ about a hundred years ago, the use of commercially produced complementary foods (CPCFs) has become commonplace in households across the world due to their long shelf-life, portability, convenience, relatively low cost, and assumed nutritional value [2]. For instance, in response to nutrient deficits, some processed cereal-based CFs are now fortified with iron. Whether the level and form of fortificants used are optimal for high-phytate cereal-based foods, CFs are not always considered by the manufacturer, in part because of the paucity of data on the bioavailability of minerals from high-phytate CFs fed to young children [7, 8].

The consumption of commercially prepared infant foods is very prevalent in many countries, exceeding the consumption of homemade foods in some situations. Although these food products may have practical advantages, there are concerns about their nutritional composition, sweet taste, bioavailability of micronutrients, diversity of ingredients, and long-term health effects. The extent to which the manufacturing, fortification, and promotion of these products are regulated by legislation varies between countries and regions [2]. The contribution of processed and ultra-processed foods in children can also be a factor that may lower the quality of the diet in children, considering that many foods exhibit unhealthy nutrient profiles, with higher amounts of sugar, trans fat, and sodium, compared with unprocessed or minimally processed foods; furthermore, after adjustment for energy intake, they may provide lower amounts of zinc, calcium, and vitamins A, B12, C, and E [9]. Looking at this in terms of child capacity, the low energy and nutrient density of the complementary foods means that large volumes of food have to be consumed to meet the infant’s requirements. This is not usually possible due to the infant’s limited gastric capacity [4].

There are very few studies in Ethiopia that are directly related to commercial infant foods, and there is a lack of studies to draw firm conclusions on whether commercially produced complementary foods (CPCFs) are mostly beneficial, nutritious, or unfavorable to infant health. **As a result, it is difficult to know how much of each nutrient is present in a given complementary food. This can make it difficult to determine whether a complementary food is providing the nutrients that an infant needs.** Given the lack of studies on complementary foods, it is important to conduct more research on this topic. This research will help to ensure that infants and young children are getting the nutrients they need to grow and develop properly. However, there is a growing trend towards the use of CPCFs in Ethiopia. This trend is likely to continue, as the country’s economy develops and more families have access to disposable income. Therefore, it is important to conduct more research on the CPCFs in Ethiopia. With this background information, the aim of the present research is to investigate the quality and safety of commercial complementary foods in relation to nutritional aspects, functional properties, labeling practices, anti-nutritional factors, and microbial load.

## Material and methods

### Sample collection

Thirty-two commercial complementary foods were purchased randomly from Addis Ababa super markets (CPCF 2, CPCF 4, CPCF 5, CPCF 6, CPCF 7, CPCF 8, CPCF 9, CPCF 14, CPCF 16, CPCF 17, CPCF 25, CPCF 26, CPCF 31, CPCF 32), and retail and small shops (CPCF 1, CPCF 3, CPCF 10, CPCF 11, CPCF 12, CPCF 13, CPCF 15, CPCF 18, CPCF 19, CPCF 20 CPCF,21, CPCF 22, CPCF 23, CPCF 24, CPCF 27, CPCF 28, CPCF 29, CPCF 30) based on the sampling method of Vella and Attard [10] and categorized based on their types.

### Laboratory analysis

#### Proximate composition determination

Moisture, ash, fat, protein, crude fiber and carbohydrate content of the commercially produced complementary foods were analyzed based on AOAC 2000 [11]. Using AOAC [11] technique 925.09, the moisture content was determined. Ash content was calculated using AOAC [11] method 941.12. The total crude fat content was measured using the AOAC [11] technique 4.5.01. Using AOAC [11] technique 979.09, the protein content was determined. The AOAC [11] method 920.169 was used to determine the crude fiber content. To calculate the percentage of total carbohydrates by difference, the AOAC [11] method 985.29 was applied.

#### Mineral analysis

The calcium, iron, zinc, manganese, and magnesium contents were determined according to the method of the AOAC [11] using flame atomic absorption spectroscopy (AAS6000). The CPCF sample was precisely weighed at 1g, and 10 mL of concentrated HNO_3_ solution was added and digested for 1 hour, yielding an almost clear solution. The digests were filtered using Whatman filter paper, and the volume of the solutions was increased using deionized water before being subjected to microwave plasma atomic emission spectroscopy. Minerals were measured, and quantification was performed using aqueous standards for calibration. The standards of the minerals used were: Zn (2–8 ppm), Ca (2.5–11 ppm), Mg (1–2.5 ppm), Mn (0.5–4 ppm), and Fe (0.5–4 ppm). Signal responses were recorded for each of the elements at their respective wavelengths: Ca (393.363 nm), Zn (213.857 nm), Mg (285.13 nm), Mn (403.07 nm), and Fe (371.993 nm).


$$Minear\;content(mg/100g)=\frac{(a-b)*v}{10w}$$ (1)

Where, W = Weight of sample in (g)

V = Volume of extract (ml)

A = Concentration of sample solution (μg/ml)

B = Concentration of blank solution (μg/m)

#### Estimated daily intake of minerals (EDI)

Estimated daily intakes (EDI) of minerals were calculated according to Zand et al. [12]. Age range, daily ration size, recommended nutrient requirements, and the contribution of human milk were considered when calculating EDI. The daily intake from the milk contribution as well as the gastric capacity of an average 8-month-old infant (30 g per kg of body weight) were taken into account to ascertain the nutritional value of these products in relation to the recommended daily intake. The gastric capacity of an 8-month old infant with an average weight of approximately 8.3 kg is estimated to be 249 g per day, which is ideal and used to calculate the contribution of CFs to the daily nutrient requirement The contribution of 600 ml of breast milk (estimated for 6–9 month infants consuming 4 times 150 ml of breast milk) for Ca = 300 mg, Fe = 7.2 mg, Zn = 4.8 mg, Mg = 38.4 mg and Mn = 1.92 mg.

### Determination of anti nutritional factors

Tannins and oxalates are among the most common anti-nutritional factors found in complementary foods, and these anti-nutritional factors were analyzed in Addis Ababa Science and Technology University, Food Science and Applied Nutrition Laboratory.

#### Condensed tannin

The method used by Rival et al. [13] was used for condensed tannin examination. A stock solution of 1000 ppm was prepared by dissolving 0.05 g of tannic acid in 50 mL of solution, which consisted of 2 mL of 10% sodium carbonate, 2.5 mL of folinciocalteu, and 45.5 mL of 70% acetone. By using the relation M1V1 = M2V2 (where M1 and M2 represent concentrations and V1 and V2 represent volume), concentrations of 1.0, 2.0, 3.0, 4.0, and 5.0 ppm were prepared from the stock solution of 1000 ppm. 0.5 g of the sample was weighed into a 100-mL bottle, followed by 50 mL of distilled water and continuous shaking for an hour using a mechanical shaker. The solution was filtered and made up to the mark in a 50 mL volumetric flask. 5 mL of the filtrate was added to a test tube with 2 mL of 0.1M FeCl_3_ in 0.1 N HCl and 0.008 M potassium ferrocyanide. The absorbance of these tannic acid concentrations was measured at 725 nm using a UV spectrophotometer (UV/JASCO V-770 spectrophotometer). A regression equation was obtained by plotting a graph of absorbance against the concentrations, which was used to determine the tannic acid content of each sample extract. The total tannic acid was expressed as mg TA equivalent/100 g of sample and calculated using the following formula:

$$CT=\frac{AT*CS}{AS}$$ (2)

Where,

CT = tannin concentration in mg/100g,

AT = absorbance of the test sample

CS = is the concentration of tannin in standard and AS is absorbance of the standard.

#### Oxalate

Oxalate content was determined using the standard method of Buta [14]. Accordingly, 2 g of flour residue was weighed into a 250-mL volumetric flask containing 190 mL of distilled water and 10 mL of 6 M HCl. The mixture was digested in a boiling water bath for 1 hour, then cooled and made up to the required consistency before being filtered. In a beaker, a 50 mL aliquot of the sample was measured, and 20 mL of 6 M HCl was added. The mixture was filtered after it had been evaporated to about half its original volume. The residue was then washed several times with warm distilled water, and 3 drops of methyl orange indicator were added to a 25-mL sample of the filtrate and titrated against a 0.05 M KMnO4 solution till a faint pink color appeared and persisted for 30 sec. The following equation was used to calculate the total oxalate content:

$$Oxalate\;content=\frac{T*Vme*Df}{ME*Mf}$$ (3)

Where:

T = Titer value of KMNO4 (ml),

Vme = v/m equivalent (1 mL of 0.05 M KMNO4 = 0.00228 g of anhydrous oxalic acid)

DF = dilution factor (VT/A that is, total volume of titrate/ Aliquot used = 2.4)

MF = mass of sample used

ME = molar equivalence of KMNO4 in oxalate concentration in g/dm3 = 5

### Determination of functional properties of commercially produced complementary foods

#### Water absorption capacity

The water absorption capacity (WAC) of flour samples was determined according to Yacoub et al. [15]. One (1) g of each flour sample was weighed and mixed with 10 mL distilled water in a 15 mL centrifuge tube. The mixture was allowed to stand undisturbed in a test tube racket at room temperature for 30 minutes and then centrifuged with a bench-top centrifuge (Centurion Scientific, Model Pro-Analytical 2004, UK) at 3000 rpm for 30 minutes. The supernatant was decanted. The flour sediment weight in each centrifuge tube was calculated. The analysis was done twice, and the results were calculated using the following formula:

The mixture was allowed to stand undisturbed in a test tube at room temperature for 30 minutes.


$$WAC=\frac{Weight\;of\;absorbed\;water}{Weight\;of\;sample}*100$$ (4)

### Rheological property

A rapid visco analyzer (RVA) model starch master (RVA 4500, Perten Instruments, Sydney, NSW, Australia) was used to determine the pasting properties of the products. Each sample was combined with 25 mL of deionized water to yield a final net weight (flour plus water) of about 28 g, which was then placed in a canister (flour weight corrected for moisture content using 3.5g at 14% moisture basis). A paddle was then inserted and shaken through the sample before the canister was inserted into the RVA. The temperature was changed at a consistent rate of 11.25°C per minute. The computer has been used to record peak viscosity, holding strength, break down, final viscosity, holding strength, set back, and pasting temperature. The experiment lasted 13 minutes, with the viscosity value being recorded every 4 seconds using thermocline software as the temperature goes up from 50 to 95°C. The rotation speed was set to 960 rpm for the first 10 seconds and then reduced to 160 rpm until the experiment ended [16].

### Microbial load of commercially produced complimentary foods

#### Determination of aerobic colony count for mold and yeast

The mold and yeast count was determined according to Bergstroem [17]. A portion of the food homogenate was mixed with a specified agar medium and incubated under specific conditions of time and temperature. It is assumed that each viable aerobic mold or yeast will multiply under these conditions and give rise to a colony. The food homogenate was prepared by transferring 10 mL of liquid sample to 90 mL of diluents or 25 g of sample to 225 mL of diluents in a flask if a shaker was used or in a sterile plastic bag. The homogenate was mixed by shaking, and 1 mL was pipetted into a tube. It was mixed carefully by aspirating 10 times with a pipette. One (1) mL was transferred from the first dilution into the second dilution tube, which contained 9 mL of the diluent, and it was mixed with a fresh pipette. This was repeated using the third or more dilutions until the required number of dilutions was made. The dilution was shaken carefully. One (1) mL of food homogenate and each of the appropriately marked duplicate dishes were pipetted into the appropriately marked duplicate dishes. 15–20 mL of the PDA were poured into a Petri dish. The prepared dishes were incubated and inverted at 370°C and 220°C for 3 days, after which the colonies were counted. If there is a growth on the negative control or if there is no growth on the positive control, the test is repeated. The average count was calculated and multiplied by the dilution. The result was expressed in cfu per g.

### Labeling practice

WHO [18], Quinn et al. [19] and Ethiopian standard [20] tools were used to examine the sample labeling procedure. The recommended labeling practices are shown in Table 2, and all of the study’s complementary foods were evaluated in accordance with these recommendations.

### Data analysis

The laboratory samples were analyzed in duplicate, and the data was presented as the mean ± standard deviation. A one-way analysis of variance (ANOVA) was performed using IBM statistics (SPSS 20.0) to assess the significant variability of the results. The means were compared by Duncan’s multiple range test, and significance was established at (p<0.05). **The PCA (principal component analysis)** was used to explore the data using data reduction, and it **was expressed using a biplot graphical method of the multivariate data matrix, which displays the two-dimensional chart** that is used to evaluate the relationship between the rows (commercially produced complementary foods) and columns (different variables). PCA was analyzed using XLSTAT 2015.1 statistical software.

## Results and discussion

### Product profile of commercially produced complementary foods

The profile of 32 CPCFs taken from the label is described on Table 1. Most of the products were cereal-based, with a majority of oats, barley, and wheat flour. Oats were found in relatively large amounts in the CPCFs (34%). CPCFs that consist of oats were CPCF1, CPCF5, CPCF6, CPCF10, CPCF11, CPCF13, CPCF14, CPCF23, CPCF24, CPCF27, CPCF28, CPCF29, and CPCF31. Next to oats, barley was the second ingredient, which was included in most of the products (34.375%). CPCF, CPCF6, CPCF13, CPCF14, CPCF21, and CPCF28 were products containing barely. There were also a few complementary foods that comprise legumes, such as chickpeas (CPCF1, CPCF5, CPCF9, CPCF13, CPCF14, and CPCF21) and soybeans (CPCF1, CPCF5, CPCF6, and CPCF31). Some of the products like CPCF 7,8,9,16,16 and 26 were premixed products. The majority of the products didn’t reveal nutritional content, and a few didn’t list the ingredients. The findings of this study have several implications for general health. First, the high prevalence of cereal-based CPCFs in the country suggests that children are not getting the recommended intake of fruits, vegetables, and legumes. This is a concern because these foods are important sources of vitamins, minerals, and fiber. And, the lack of nutritional information on most of the CPCFs makes it difficult for parents to make informed choices about which products to feed their children. This could lead to children consuming CPCFs that are not nutritionally adequate. In order to address these concerns, the regulatory bodies should take steps to ensure that CPCFs are more nutritious and that parents have access to information about the nutritional content of these products. The regulatory bodies could also require CPCF manufacturers to list their ingredients on the product label. These measures would help to ensure that children are getting the nutrients they need to grow and develop properly.

**Table 1:** Profile of the commercially produced complementary foods as found from the labels (N = 32).

No	Code	List of ingredients	State of the CPCF	Premixed or not
1	CPCF1	Barley, Oats, Wheat, Corn, Rice, Red *Teff*, Sorghum, Chickpea, Lentil, Soybean, Linseed, Sunflower seed and Fenugreek	Powdered	-
2	CPCF2	Corn starch	Powdered	-
3	CPCF3	Plain *shiro*	Powdered	-
4	CPCF4	Cavendish banana	Powdered	-
5	CPCF5	Barley, Oats, Corn, Rice, Sorghum, Chickpea, Lentil, Soybean, Bean, Linseed, Sunflower seed and Fenugreek	Powdered	-
6	CPCF6	Oats, Soybean, Brown lentil, Red *Teff*, Brown Wheat, Corn, Peanut, Barley and Bean	Powdered	-
7	CPCF7	Wheat flour, Corn,	Powdered	Premixed
8	CPCF8	Wheat flour, rice, corn, skimmed milk powder, sugar, iodized salt, vegetables (spinach, carrot, peach),vanilla.	Granulated	Premixed
9	CPCF9	Wheat flour, soya flour, Chickpea flour, full fat milk powder, iodized salt & flavor	Powdered	Premixed
10	CPCF10	Oats, Red Teff, Fenugreek, beans, Sorghum, lentil	Powdered	-
11	CPCF11	Oats	Powdered	-
12	CPCF12	Oats	Powdered	-
13	CPCF13	Sunflower, Barley, Oats, Wheat flour, Red Teff, Chickpea, Flaxseed, Beans, Fenugreek, soya bean, sorghum	Powdered	-
14	CPCF14	Barley, Oats, Red Teff, Roasted barley, Chickpea, Flaxseed and Sesame	Powdered	-
15	CPCF15	Wheat flour, Sugar, Iodized salt, Vanilla	Granulated	Premixed
16	CPCF16	Wheat flour, Soya flour, skimmed milk powder, Palm oil, Fruit pulp (Banana, Orange, Pineapple, Mango, Strawberry), Sugar, Iodized salt, Vanilla.	Granulated	Premixed
17	CPCF17	Wheat flour, corn flour, Powdered milk, Sugar and Iodized salt and Vanilla.	Powdered	-
18	CPCF18	Bula	Powdered	-
19	CPCF19	Barley	Powdered	-
20	CPCF20	Barley	Powdered	-
21	CPCF21	Chickpea, Flaxseed, Sorghum, Barley, Corn flour, Sesame and brown wheat	Powdered	-
22	CPCF22	Barley	Powdered	-
23	CPCF23	Barley, Oat	Powdered	-
24	CPCF24	Barley, Oat	Powdered	-
25	CPCF25	Wheat flour, Milk powder, Soya powder, Sugar, Vanilla and Iodized salt.	Powdered	
26	CPCF26	Wheat flour, rice, corn, skimmed milk powder, sugar, iodized salt, Fruit pulp (Fruit Cocktail), vanilla.	Granulated	Premixed
27	CPCF27	Oats	Powdered	-
28	CPCF28	Oats and barley	Powdered	-
29	CPCF29	Oats	Powdered	-
30	CPCF30	Bula	Powdered	-
31	CPCF31	Soybean, Oats, Lentil, Red Teff, Sorghum, Chick pea, Peanut, Fenugreek, Wheat, flaxseed, Corn, Bean, Sesame, Bula and Oats	Powdered	-
32	CPCF32	Oats and barley	Powdered	

CPCF: Commercially Produced Complementary Foods

### Labeling practice commercially produced complementary foods

Table 2 shows the information on the packaging based on criteria that were divided into three main categories, namely, information that must be on the packaging, TO DO, and NOT TO DO [19]. The first category was related to information that must be included on the packaging, and based on this parameter, all of the products had ingredients on their packaging, and 90.63% had the producer’s name. However, nutritional composition (37.5%), energy (31.25%), instructions for appropriate preparation and use (37.5%), instructions for safe and appropriate storage (40.62%), expiration date (37.5%), and the producer’s address (31.25%) were stated in less than 50% of the CPCFs. The second category was the "TO DO" list that must be displayed in the package. As stated in Table 2, only 6.3% of the CPCFs had a label that encouraged continued breastfeeding up to 2 years and beyond, despite the mandate stated by WHO [18]. The specification of the appropriate age of introduction for complementary foods was only labeled on 31.25% of the products. According to Ethiopian standard [20], CPCFs package should contain a warning that indicates list of allergy causing ingredients if they contain one of these ingredients: cereals containing gluten, peanuts, soybean, nuts etc. However, none of the products had this warning even though there were products that contained the aforementioned ingredients. According to WHO [18], a CPCF package should include a warning that it cannot be used in place of breast milk, but only 21.88% of CPCFs have that information on the label. The third topic of labeling practice assessment was the "NOT TO DO" list. This category requires that the packages of the products state an age of introduction less than 6 months, but none of the products have displayed misleading allegations.

**Table 2:** Criteria used to evaluate the adequacy of the labelling of CPCFs marketed as complementary foods (N = 32).

	Recommendations Figuring in the Codex Stan 074 Rev. 2006	Number of Products which fulfilled the standard out of 32 products (%)
**Information that MUST be on the packaging**	Ingredients	100%
	Nutritional composition^1^ and energy^2^	37.5%^1^, 31.25%^2^
	Provides instructions for appropriate preparation and use	37.5%
	Provides instructions for safe and appropriate storage	40.62%
	Expiration date	37.5%
	Producer’s name	90.63%
	List of allergy causing ingredient	0%
	Date of minimum durability	0%
	Producer’s address& country of origin	31.25%
	Recommendations Based on the International Code and WHA^*^ **Resolutions**	**Number of Products which fulfilled the standard Out of 32 (%)**
**TO DO**	Proposes a daily ration per serving	31.25%
	Specifies an appropriate age of introduction (from 6 months)	31.25%
	If pictures are permitted by national laws, pictures of babies must show babies older than 6 months (with physical or developmental milestone reached after 6 months)	100%
	States the importance of exclusive breastfeeding till 6 months	21.88%
	Encourages continued breastfeeding up to 2 years old and beyond	6.2%
	With the indication ‘‘Can Not replace breast milk”	21.88%
**NOT TO DO**	States an age of introduction less than 6 months	0%
	Mentions misleading allegations	0%

Source: Quinn et al. 2018, *WHA-World Health Assembly, 2016

### Proximate composition of commercially produced complementary foods

#### Protein content

The proximate composition of 32 commercially produced complimentary foods is presented in Table 3. The range of protein values varied from 0.92±0.70–18.16±0.3 g/100g. Products such as CPCF2, 18, 30 and 31 (p>0.05) had the lowest value, and CPCF 3 had the largest value. There is a significant difference between the CPCFs (p<0.05). Comparatively, the findings of Dimaria *et al*. [21] indicated that the protein content of CPCFs ranged from 9.2–21.8 g/100g, which had higher range than the current study [21]. The variation in the protein content can be attributed to the types of ingredients. The lowest protein content was observed in commercially produced complementary foods consisting of corn starch, “bula” and banana as main ingredients. Most of the samples collected contained one to several cereals or some legumes as sources of protein, such as chickpea and soybean. According to WFP [22], complementary foods need to have 16 g/100g protein; however, none of the products have met this value. Conversely, other standards, like Codex Alimentarius commission, [23], set that the protein content of complementary foods should fall between 6–15 g/100g and based on this, 25 samples were aligned with the CODEX specification, 6 products were below the recommended range, and one product exceeded the recommendation level. The fifth Nordic Nutrition recommendation indicates that 15% of protein energy is proposed as the upper limit at 12 months, and when compared to this study, it shows that one of the 32 products has exceeded the upper limit [1].

**Table 3:** Proximate composition (Mean±SD) of commercially produced complementary foods (g/100g DW).

Treatment	Moisture	Ash	Fat	Protein	Crude fiber	COH	Calorie (kcal)
CPCF1	3.48±0.43^jklm^	2.33±.00^abcdef^	6.13±.36^b^	12.81±0.41^c^	2.34±.06^i^	72.91±.20^i^	398.01±.39^ab^
CPCF2	11.00±1.13^c^	0.80±.00^ij^	.63±.18^i^	.92±0.30°	3.45±.04^b^	86.66±.46^b^	355.93±5.41^m^
CPCF3	7.19±0.28^de^	6.67±.00^a^	4.19±.01^e^	18.16±054^a^	2.49±.01^h^	63.80±.64^j^	365.50±.648^kl^
CPCF4	7.15±0.21^def^	3.17±0.23^b^	1.00±.35^hi^	2.67±0.31^n^	13.20±.10^a^	86.02±.45^bc^	363.72±1.85^lm^
CPCF5	4.89±0.42^hi^	1.84±0.23^efghi^	3.10±2.11^d^	7.90±0.89^jk^	3.15±.05^d^	81.39±2.26^ef^	393.06±9.82^cde^
CPCF6	5.88±0.70^efgh^	1.84±0.23^efghi^	5.56±.62^bc^	12.70±0.50^c^	2.53±.03^gh^	74.03±0.13^hi^	396.95±6.82^b^
CPCF7	3.40±0.28^klm^	1.00± .00^hij^	1.63±.18^ghi^	10.16±0.37^efg^	1.64±.00^l^	83.82±.06^cde^	390.53±.25^cdef^
CPCF8	0.80±0.28^n^	1.50±.14^efghij^	1.57±.26^ghi^	10.01±0.70^fgh^	2.80±.04^e^	83.34±.37^de^	387.41±1.64^defgh^
CPCF9	7.50±0.14^d^	2.67±.00^bcd^	6.30±.26^b^	10.25±0.007^ef^	3.33±.02^bc^	73.30±.33^e^	390.80±.73^cdef^
CPCF10	5.70±0.98^fgh^	2.17±.23^bcdefg^	.94±.09^hi^	11.26±0.43^de^	3.32±.02^bc^	79.95±.63^fg^	373.22±4.46^jk^
CPCF11	5.50±0.99^ghj^	1.50±.24^efghi^	2.81±.08^ef^	8.63±0.37^ij^	2.63±.03^fg^	81.56±.25^def^	386.06±3.46^defghi^
CPCF12	4.00±0.00^hijkl^	1.67±.47^efghi^	2.00±.18^efgh^	5.96±0.31^l^	3.11±.01^d^	86.38±.06^b^	387.33±2.77^defgh^
CPCF13	5.10±0.71^ghi^	2.00±.00^cdefgh^	1.44±.18^ghi^	10.12±0.24^efgh^	2.42±.02^i^	81.35±.09^e^	378.79±4.15^hij^
CPCF14	7.00±0.00^def^	2.44±1.75^bcd^	.82±.01^i^	9.98±1.61^fgh^	3.33±.03^bc^	79.78±1.13^fg^	366.33±6.53^kl^
CPCF15	0.03±0.00^n^	2.67±.00^bcd^	1.37±.00^hi^	10.16±0.36^efg^	5.20±.10	85.77±1.14^bc^	396.09±.00^b^
CPCF16	4.50±0.71^hijk^	1.00±.00^bhij^	4.9±.45^cd^	11.13±0.25^def^	3.12±.02^d^	75.58±1.06^h^	388.98±.53^cdefg^
CPCF17	2.60±0.00^m^	2.67±.00^bcd^	.75±.17^i^	6.92±0.09^kl^	3.92±.02^a^	83.16±.04^de^	367.02±.80^kl^
CPCF18	13.30±0.14^b^	0.80±.00^ij^	1.76±.53^ghi^	1.29±0.44°	1.04±.01^n^	82.86±.54^de^	352.35±2.09^m^
CPCF19	9.90±0.42^c^	0.60±.28^j^	.63±.18^i^	13.09±0.31^c^	2.11±.01^k^	75.79±.94^h^	361.13±1.94^lm^
CPCF20	0.05±0.00^n^	2.17±1.18^bcdefg^	1.32±.26^hi^	7.40±0.02^k^	2.33±.03^ij^	89.08±.87a	397.70±6.05^b^
CPCF21	4.90±0.71^hi^	2.67±.00^bcd^	8.19±.45^a^	8.63±0.37^ij^	2.41±.01^i^	75.63±.21^h^	410.67±5.04^a^
CPCF22	5.70±0.99^fgh^	2.17±.23^bcdefg^	1.13±.36^hi^	7.01±0.31^k^	2.41±.01^i^	84.00±1.21^cd^	374.16±3.13^jk^
CPCF23	6.50±0.42^defg^	1.34±.47^fghij^	2.94±.27^e^	9.15±0.44^ghi^	2.61±.01^fgh^	80.08±1.47^fg^	383.35±2.26^efghi^
CPCF24	5.70±0.14^gh^	1.17±.23^ghij^	1.76±.18^fghi^	10.99±0.77^def^	2.21±.01^jk^	80.40±.99^fg^	381.28±.62^ghij^
CPCF25	2.80±0.57^lm^	3.00±.47^bc^	2.50±.18^efg^	3.96±0.06^m^	3.12±.03^d^	87.75±1.63^ab^	389.30±5.03^cdefg^
CPCF26	4.80±0.71^ehij^	2.17±.23^bcdefg^	6.44±.62^b^	10.91±0.06^def^	3.12±.01^d^	75.70±.39^h^	404.32±6.86^a^
CPCF27	3.40±0.85^klm^	1.67±.47^efghi^	2.75±.35^ef^	9.02±0.50^hi^	2.33±.04^ij^	83.17±.23^de^	393.48±.26^bcde^
CPCF28	5.50±1.27^gh^	2.17±.23^bcdef^	2.88±.18^e^	11.21±0.50^de^	2.61±.01^fgh^	78.25±.37^g^	383.70±5.03^efghi^
CPCF29	5.95±1.20^efg^	1.00±.00^hij^	1.19±.08^hi^	8.67±0.5^ij^	3.20±.14^cd^	83.20±1.79^d^	378.12±4.37^ij^
CPCF30	17.30±1.14^a^	0.60±.28^j^	1.75±.54^fghi^	1.05±0.12°	1.20±.14^m^	79.30±1.07^fg^	337.15±.95^n^
CPCF31	4.50±1.14^hijk^	1.33±.00^fghij^	6.38±.36^b^	1.10±0.06°	3.13±.04^d^	86.70±.43^b^	408.54±1.20^a^
CPCF32	7.00±0.00^def^	2.17±.23^bcdefg^	1.13±.17^hi^	11.56±0.25^d^	2.73±.04^ef^	78.15±.17^g^	368.96±.059^kl^

Values are reported in mean ± SD. Means not sharing a common superscript letter across the column are significantly different (P<0.05). CPCF: Commercially Produced Complementary Foods

#### Fat content

Table 3 shows the range of fat composition of the CPCFs, and the results varied from 0.63±0.18 to 8.19±.45 g/100g. CPFCF 2, 4, 7, 8, 10, 13, 14, 15, 17, 18, 19, 20, 22, 24, 29, 30, and 32 had the lowest values, which did not have a significant difference (p>0.05). CPFCF 21 had the largest value, which is significantly different from the other CPCFS (P<0.05). The fat contents were below, and often far below, the level recommended in the Codex Alimentarius specifications in all of the 32 CPCF samples. Similar results were observed regarding the fat content in a research study conducted in four African countries, in which 24 out of 32 samples failed to meet the Codex Alimentarius specification [23]. However, more than half of the CPCFs are consumed after cooking, which may require additional oil thus the fat content might be enhanced during the cooking process. Low fat content, below the Codex recommendation, was also observed previously, and it is consistent with the report of Gibbs et al. [24]. Codex Alimentarius commission [23] and WFP [22] specifications suggested that a complementary food should contain 9 g of fat per 100g of food, and all of the CPCFs fall under this standard. Lutter & Dewey [25] recommended higher values than the aforementioned standards, which is 12.7 g/100g; nonetheless, all of the CPCFs were much lower than this value too. Producers should, thus, increase the proportion of oilseeds or healthy fat alternatives, although the impact on the conservation of the product would need to be evaluated and could require reducing the recommended maximum storage time.

#### Crude fiber

The fiber content of the CPCFs is listed in Table 3. The results ranged from 1.04±0.01 to 13.20 ±0.01 mg/100g. CPCF 18 had the lowest value, and CPCF 4 had the largest value. There is a significant difference (P<0.05) between these products. Mekuria et al. [26] reported 2.75 ± 0.17 g/100 g of fiber content, which is comparable with the majority of the current study. According to Codex Alimentrius (CA), the fiber content of commercial complementary foods should be <5 g/100g [23]. Except for one product, the rest of them fall under CA requirements. The higher amount of fiber than recommended can be attributed to the use of a larger amount of unhulled cereals [27, 28], and 96.87% of the CPCF products have met this requirement in the present report.

#### Energy content

The energy content of the CPCFs are listed in Table 3. The results ranged from 337± 0.95 to 410±5.04 Kcal/100g. CPCF 21, 26, and 31 had the highest Kcal, while CPCF 30 had the lowest. There is significant variation (P <0.05) between the CPCFs. The lower energy content of the samples may explain the lower fat and protein content observed during this study. Sizeable numbers of the dry cereal products were below the 410 kcal/100 g energy density level [22]. Only three products met the requirement.

#### Moisture content

The moisture contents of the CPCFs are listed in Table 3, and the products ranged from 0.03±0.00 to 17.3±0.14 g/100g. CPCF 30 had the highest moisture content, while CPCFs 15, 8, and 20 had the lowest. There was a significant difference (p<0.05) in moisture content between the higher and lower groups of CPCFs. CPCFs (43.75%) were in line with the Codex standard [23], which states that the moisture content of complementary products should be 5% or less. The highest moisture content (17.4%) was obtained from a CPCF that contains “Bula” as a main ingredient, high moisture contents in food samples encourage the growth of microorganisms; hence it leads food spoilage.Moisture content determination is key factors affecting the storage, shelf life, and safety of foods. Some of the highest moisture content could be attributed to improper drying of the raw materials before milling or improper storage of products at high humidity [27]. The lowest was from CPCFs that were formulated from cereals, dried milk, and dried vegetables.

#### Ash content

Table 3 describes the ash content of the products, ranging from 0.6±0.28 to 4.67±0.47g/100g. CPCFs such as 19, 2, 7, 8, 16, and 18 had the lowest ash content, and CPCF 3 had the highest ash content. There is a significant difference (p<0.05) the CPCFs. WHO/FAO [29] recommended that the ash contents of complementary foods should be less than 5 g/100g, and all the CPCFs except one (CPCF3) were up to the standards. The lowest result was from a barley mix complementary food, and the highest (4.67%) was from a complementary food produced from chickpeas. According to the Codex Alimentarius, the ash content should be less than <3%, and based on this requirement, 29 products met the standards; the other 2 (CPCF4 and CPCF25) were slightly higher, and only one product had a higher variation from the standard (4.67% in CPCF3). The variations in ash may be resulted from processing techniques such as dehulling, roasting, and milling, given that most minerals are concentrated in the outer layers of the grains [27].

#### Carbohydrate

The carbohydrate content of the products are listed in Table 3. The results ranged from 63.80 ± 0.64 to 87.75 ± 1.63 g /100g. CPCF 1, 2, 3, 9, and 13 were significantly different from the other products (P<0.05). The recommended value of carbohydrate in commercial complementary foods is > 60% [23]. Based on this, it can be concluded that all of the CPCFs had met the requirement. Total carbohydrate content is highly affected by the percentage of protein, fat, and fiber in the particular food. Low fat, protein, and fiber content results in high total carbohydrates in the food. The high carbohydrate contents of complementary foods obtained in this study could be attributed to the inclusion of large proportions of cereals in formulations other than legumes and other non-cereal foods [27].

### Mineral composition of commercially produced complementary foods

#### Iron content

The mineral profile of the CPCFs is presented in Table 4. The iron content of the samples ranged from 1.38±0.04 to 15.10±4.45 mg/100g. CPCF 8, 9, and 31 were significantly different (P<0.05) from the other CPCFs. The present study has different results when compared to the results of some countries, like Peru (0.4 mg/100 g), Ghana (1.2 mg/100 g), and Bangladesh (0.4 mg/100g) [30]. When compared to the standards from FAO and IOM, the iron content obtained from commercially produced complementary foods in the respective countries shows that the products didn’t meet the requirement. Even though some of the products claim that they are fortified with premixes, the results showed otherwise. According to Agbemafle et al. [31], the iron content of a wean mix made of cereal products is 0.77± 0.01 mg/100g, which is significantly lower than the iron content of all of the CPCFs reported in this study. The iron contents of the present studies show some similarity with the report of Amare et al. [32], in which the iron contents varied from 5.85–22.31 mg/100g. WHO recommendations, as stated by Dewey and Brown [25], indicated that 14 mg/100g must be provided from commercial complementary foods [25]. Based on this, 12.5% of the CPCFs qualified for this standard. GAIN Ethiopia’s manual for the manufacture of complementary foods recommends up to 7–11 mg/100g. This indicated that 73.7% of the products didn’t align with the standards, and only 26.3% of the products had met the stated value as determined by GAIN [33]. In addition, the Codex Alimentarius [23] standard recommends that a complementary food contain 11.6 mg/100 g of iron. The variation of the samples with this standard was also analyzed, and it indicates that eleven products are below this standard. The lowest Fe concentration was observed in a CPCF composed of cereal products like oats, flaxseed, and beans. The highest value was from a product that contains soybean and wheat as main ingredients.

**Table 4:** Mineral composition of commercially processed complementary foods (mg/100g) and estimated dietary intake of minerals (mg/day).

Treatment	Iron	Zinc	Calcium	Magnesium	Manganese	EDI Fe	EDI Zn	EDI Ca	EDI Mg	EDI Mn
CPCF15	14.13±.32^abcd^	6.78±.25^cde^	42.65±2.19^f^	16.85±.07^def^	1.30±.00^cde^	42.37±.79^abcd^	21.67±0.62^cde^	839.58±15.34^cde^	80.36 ±.18^def^	5.16±0^bc^
CPCF25	14.35±.14^defg^	5.03±.18^cd^	30.55±.78^g^	14.45±.07^cde^	1.27±.04^abc^	42.80±.18^defg^	17.33±0.46^cd^	731.40±11.41 ^cd^	74.38±.18^cd^	5.09±0.09^ab^
CPCF26	14.25±3.25^efg^	6.75±.14^i^	79.60±6.36^fg^	17.10±.28^m^	1.40±.21^e^	42.73±8.15^efg^	21.61±0.35^h^	838.03±8.76 ^h^	80.98±.70^m^	5.41±0.53^c^
CPCF5	12.55±.28^abc^	3.85±.35^ab^	199.85±3.04^i^	28.60±.28^g^	1.55±.00^abcde^	38.45±.70^ab^	14.77±1.43^ab^	667.83±35.51^ab^	109.61±.70^g^	5.78±0^abc^
CPCF1	14.98±2.02^abc^	0.45±.64^ab^	213.60±16.40^k^	24.65±.21^g^	3.00±.28^bcde^	44.49±5.02^abc^	13.39±1.58^ab^	633.42±39.46^ab^	99.78±.53^g^	9.39±0.70^abc^
CPCF8	4.65±1.77^abcd^	2.10±.00^cdef^	212.40±1.27^d^	6.65±.21^b^	1.78±1.31^abcde^	18.78±4.4^abcd^	10.03±0.00^cde^	549.72 ±.00^cde^	54.96±.53^b^	6.34±3.26^abc^
CPCF10	2.05±.28^ghi^	1.55±.35^fghi^	215.85±3.89^c^	3.44±3.20^j^	.44±.51^abcde^	12.31±.70^ghi^	8.66±0.88^fgh^	515.62±21.92^fgh^	46.97±7.96^j^	3.02±1.26^abc^
CPCF9	9.53±2.02^a^	7.73±.04^cdefg^	364.45±10.81^c^	22.80±.28^d^	.66±.84^a^	30.92±5.02^a^	24.04±0.09^cdef^	898.48±2.19^cdef^	95.17±.70^de^	3.56±2.01^a^
CPCF11	1.68±.32^hi^	.68±.04^hi^	59.90±12.02^c^	1.20±.00^j^	1.03±1.31^de^	11.37±.79^hi^	6.48±0.08^gh^	461.37 ±2.19^gh^	41.39±.00^k^	4.47±3.26^c^
CPCF14	11.70±1.77^hi^	4.03±.32^i^	136.15±.21^j^	23.95±.07^lm^	1.10±1.41^cde^	36.33±4.41^hi^	14.82±0.79^h^	669.07 ±19.73^h^	98.04±.18^l^	4.66±3.52^bc^
CPCF16	9.05±2.05^def^	4.15±.49^cde^	115.25±.63^g^	23.60±.14^ef^	2.03±.11^abcd^	29.73±5.11^def^	15.14±1.24^cde^	676.82±30.69^cde^	97.16±.35^ef^	6.96±0.26^ab^
CPCF17	2.28±1.80^hi^	.45±.08^i^	197.95±.35^de^	9.65±.14^i^	1.32±1.52^abcde^	12.87±4.49^hi^	5.91±0.19^h^	447.11±4.82^h^	62.43±.18^i^	5.22±3.79^abc^
CPCF31	15.10±4.45^i^	.82±.14^ghi^	186.55±1.91^g^	26.90±.00^kl^	.80±.00^e^	44.80±11.09^i^	6.84±0.35^fgh^	470.36±8.77^fgh^	105.38±.00^kl^	3.91±0^c^
CPCF2	10.10±.21^ab^	3.02±2.80^bc^	95.25±.35^l^	22.30±.42^h^	2.88±.67^bcde^	32.35±.52^ab^	12.32±6.97^bc^	606.76±173.6^bc^	93.93±1.06^h^	9.08±1.67^abc^
CPCF19	5.50±2.69^cde^	2.75±1.34^defgh^	60.10±.42^h^	8.70±.00^f^	1.80±.00^ab^	20.90±6.70^cde^	11.65±3.34^defg^	590.02±83.30^defg^	60.06±.00^f^	6.40±0^a^
CPCF21	10.70±.07^fgh^	5.10±.85^efgh^	323.40±.42^j^	34.20±.14^i^	1.00±.42^abcde^	33.85±.18^fgh^	17.50±2.10^efg^	735.73±52.6^efg^	123.56±.35^i^	4.41±1.06^abc^
CPCF23	8.48±.67^abcd^	4.65±.07^bc^	114.90±.56^b^	25.45±.07^a^	2.23±.60^cde^	28.31±1.68^bcd^	16.38±0.18^bc^	707.83 ±4.38^bc^	101.77±.18^a^	7.46±1.5^bc^
CPCF18	6.50±1.20^a^	.88±.74^i^	126.75±.92^e^	.00±.00^c^	.30±.00^cde^	23.39±2.99^a^	6.98±1.85^h^	473.77 ±46.03^h^	Nd±.00^c^	2.67±0^bc^
CPCF13	1.38±.04^def^	1.9±0.0^a^	122.6±1.27^a^	2.1±0.14^f^	0.20±0.00^cde^	10.63±.09^def^	9.53±0.00^a^	537.32 ±.00^a^	43.63±.35^f^	2.42±0^bc^
Standard	14	4–5	500	168	1.3	8.7	7	700	100	3

Values are reported in mean ± SD. Means not sharing a common superscript letter across the column are significantly different (P<0.05). CPCF: Commercially Produced Complementary Foods.

Mineral composition of commercially processed complementary foods and estimated dietary intake of minerals for 6–9 months

Ca = 50mg/100ml, Fe = 11.2mg/100ml,Zn = 0.8mg/100ml, Mg = 6.4mg/100ml of breast milk was used to calculate EDI.

#### Zinc

The zinc content of the individual CPCFs is presented in Table 4. It ranged from 0.64±0.45 to 6.78±0.25mg/100g. Few products (CPCF 13) were significantly different from the rest of the products (P<0.05). A similar report on commercial weaning mixes in the UK found that the zinc content of CPCFs is 0.34±0.10–0.54 ±0.14 mg/100 [15], which is within the same range as the current study. The least amount of zinc content was detected in a winning mix of the main ingredient, Bula. According to the WHO [4], a CPCF should provide 4–5 mg per serving. WFP also claims that the maximum zinc content can reach 14 mg/100g [22]. Most of the CPCFs met the recommended level of 4–5 mg of Zn in CFs for IYCF (4).

#### Calcium

Table 4 displays the calcium content of the CPCFs. The contents ranged from 30.55±0.78 to 364.45±10.82 mg/100g. CPCF 5, 8, 13, and 2 were significantly different (p<0.05) from the other products. The calcium content of commercial complementary products ranged from 17.4–56.4 mg/100 in a UK-based report [15], which was lower than the current study. According to the WHO, the calcium content of complementary foods should range from 100 to 200 mg per serving [29], in which the serving size is 50 g, and this shows that 73% of the CPCFS in this study didn’t meet the required amount of calcium content. The Codex Alimentarius commission [23] standard states that CFs should contain 500 mg/100 g of calcium. According to this requirement, all of the products didn’t meet the Codex specification. GAIN Ethiopia has also set standards of 250–500 mg/serving, where 89.5% of the CPCFs in this study didn’t meet the specified standard. Anuonye et al. [34], who developed a sorghum-soybean-with-sardines composite complementary food with a calcium content of 77.57–272.37 mg/100g, which has some differences and some similarities with the findings of this study. Ajala et al. [35] found 441 mg/100 g, while the current study found a maximum of 364.45±10.82 mg/100g. Masunzu [27] reported Ca content in commercially produced complementary foods in the range of 59.56 to 145.45 mg/100 g.Accordingly, it is necessary to work on satisfying the standards of WHO, CODEX, and GAIN Ethiopia.

#### Magnesium

The magnesium content of the complementary foods is presented in Table 4, where the values ranged from 1.2±0 to 34.2±0.14 mg/100g. Almost all of the magnesium contents of the CPCFs had a significant difference (p<0.05) from each other except CPCFs 15, 16, and 25. In comparison to our study, Ajala et al. [35] found a higher magnesium content of 96.09 mg/100g. The magnesium content was lower than that reported by Codex Alimentarius commission [23]. WFP [22] recommends 168 mg/100 g, and Codex Alimentarius commission [23] sets 60 mg/100 g. In general, the magnesium content of the CPCFs is very low, and none of the CPCFs has met these standards.

#### Manganese

The manganese content of the complementary foods is presented in Table 4. Manganese concentrations ranged from 0.80±0.00 to 3±0.28 mg/100g. The majority of the products are not significantly different (p>0.05) from each other. The manganese results are similar to the report of Diamara et al. [20], but differ from Ajala et al. [35]. According to Codex Alimentarius commission [23], the manganese content of complementary foods should contain 1.3 mg per 100 g, and 36.8% of the CPCF didn’t meet this standard.

### Estimated daily intake of minerals

Table 4 shows the estimated daily mineral intake for children (aged 6–9 months), including breast milk. The EDI method is used to calculate the amount of minerals that an infant should consume per day. The EDI is calculated using the calcium (50 mg/100 mL), iron (1.2 mg/100 mL), zinc (0.8 mg/100 mL), and magnesium (6.4 mg/100 mL) contents of breast milk [12]. The estimated daily intake of the iron, which is contributed from breast milk, gastric capacity of the infants and the CPCFs ranged from 10.63 to 44.80 mg/day. The estimated daily intake of zinc content from the CPCFs was in the range of 5.91 ± 0.19–21.67 ± 0.62 mg/day (Table 4). The levels indicated that there is excessive intake of minerals in some of the products; only four of the CPCFs have shown results similar to the standard (8.7 mg/day) set by FAO/WHO [29]. The other products (78.94%) had shown higher results when compared to the standard. It can be concluded that the products impart an excessive daily intake, however, plant based complementary foods have components that inhibit bioavailability of minerals [36] thus, even though the result showed excessive daily intake, the character of such kinds of CFs (low bioavailability) should be considered since all of the CPCFs in the present studies are plant derived CPCFs. The present study designated higher results when compared to prior studies [15, 37]. However, both of these results are higher than the standard 8.7 mg/day [22], they are still lower than the present studies.

Six products (31%) didn’t show a higher difference with the FAO/WHO [29] standard (7 mg/day). The level of zinc EDI in the rest of the CPCFs was higher than for infants in this age range. It is important to note that excessive iron and zinc intake can have a negative impact on other minerals, such as copper [15]. The EDI of the calcium content of the CPCFs ranged from 515.62 ± 21.92–735.73 ± 52.63 mg/day. The contribution of calcium to daily intake shown in the table indicated that 57.89% of the product met the standard set for calcium, which is 700 mg/day [22]. However, the majority of the other products had lower results and didn’t fall under the recommended setting. The magnesium EDI ranged from 41.39 ±0 to 123.56±0.35 mg/day, which had a significant difference when compared to the other products, which fall short of the 100 mg/day requirement [29]. The present study showed a lower result when compared to Ajala et al. [35], where the EDI of magnesium was recorded as 96–131 mg/day. The Manganese EDI ranged from 2.42±0 to 9.08±1.68 mg/day and the results were not significantly different from each other. The variation among the products can be attributed to the disparity and ratio of the ingredients and their types.

### Mineral interrelationship

The interrelationship of minerals is shown in Table 5. Zn/Fe and Ca/Mg interrelationships ranged from 0.01–0.48 and 2.11–62.75, respectively. Evaluating the mineral profiles of food samples is not enough to predict their bioavailability; their interrelationships, which are an indicator of their bioavailability, should be intricately taken into consideration. The ratio is calculated by dividing the concentration of the first mentioned element by that of the second mentioned element [38]. Several mineral ratios may work together to contribute to mineral imbalances. Watts [39] noted that the interrelationship of mineral elements is more important than knowing the mineral contents alone. Hence, the ratios of some elements relative to others are of paramount importance. Zn/Fe and Ca/Mg ratios are used to describe mineral interrelationships in infant diets. WHO recommendations for the Ca/Mg ratio range from 3–11 for a good infant diet [38]. As indicated in Table 5, the results of the Ca/Mg ratio range from 2.11 to 58.38. A markedly elevated Ca/Mg ratio is associated with increased insulin levels [33]. According to the findings of this study, the Ca/Mg ratios of half of the CPCFs did not meet the recommended standard (3–11). WHO [40] recommendations for Zn/Fe ratio range from 0.8 to 3.5 for a good infant diet, and Table 5 reveals that none of the CPCFs have fallen under the recommended range. Therefore, in terms of Zn/Fe, the products can be considered poor CFs with regards to the standard of the WHO [40].

**Table 5:** Zinc/iron and calcium/magnesium relationship.

Treatment	Zn/Fe	Ca/Mg
CPCF1	0.48	2.53
CPCF2	0.04	2.11
CPCF3	0.05	4.65
CPCF8	0.03	6.99
CPCF9	0.03	8.67
CPCF10	0.05	31.94
CPCF11	0.08	62.75
CPCF13	0.09	15.98
CPCF14	0.04	49.92
CPCF15	0.04	5.68
CPCF16	0.05	4.88
CPCF17	0.02	20.51
CPCF18	0.01	6.93
CPCF19	0.03	4.27
CPCF21	0.06	6.91
CPCF23	0.05	9.46
CPCF25	0.06	4.51
CPCF26	0.01	-
CPCF31	0.15	58.38

Zn/Fe = zinc to iron ration; Ca/Mg = calcium to magnesium ratio. CPCF: Commercially Produced Complementary Foods

### Anti-nutrient content of commercially produced complementary foods

#### Tannin content

The tannin content of the commercial complementary foods is listed in Table 6. The tannin content ranges from 49.2±1.27 to 90.09±0.47 mg/100g. With a few exceptions, the results differ significantly (P<0.05). The maximum tolerable level of tannin should be 560 mg/100g [41], and all of the tannin contents of the CPCFs were lower than this specification. Despite not exceeding the maximum tolerable level, the products had a higher tannin content when compared to Keyata et al. [42] & Gemeda et al. [41]. The tannin content of commercial mix reported by Mekuria [26] was 63.69±0.34 mg/100g. Cereal mix contains a high level of tannin due to the characteristics of their ingredients [27]. Tannins usually affect protein digestibility and lead to reduction of essential amino acids by forming reversible and irreversible tannin-protein complexes between the hydroxyl group of tannins and the carbonyl group of proteins [43] and to reverse this effect, different traditional methods and technological processing ways such as soaking, milling, debranning, roasting, cooking, germination and fermentation can be used for reducing these anti-nutritional components in complementary foods [44].

**Table 6:** Anti-nutritional content of commercially produced complementary foods (mg/100g DM).

Treatment	Oxalate	Tannin
CPCF1	12.19±1.77^defg^	57.45±1.77^k^
CPCF2	5.47±.00^g^	65.05±2.33^ij^
CPCF3	30.1±3.87^a^	64.15±.07^j^
CPCF4	10.94±.00^ef^	49.20±1.27^l^
CPCF5	21.89±.00^bc^	81.55±.49^cd^
CPCF6	16.42±.00^cde^	52.00±2.69^kl^
CPCF7	5.47±.00^g^	79.75±2.05^de^
CPCF8	16.42±.00^cde^	99.05±.78^a^
CPCF9	19.16 ±3.87^bcd^	65.90±1.13^ij^
CPCF10	10.94± 00^ef^	72.40±.14^f^
CPCF11	16.42±.00^cde^	99.95±.49
CPCF12	16.42±.00^cde^	68.13±1.17^ghi^
CPCF13	13.68±3.87^def^	79.18±1.34^de^
CPCF14	5.47±.00^g^	70.25±.21^fgh^
CPCF15	24.63±3.89^ab^	77.04±.76^e^
CPCF16	8.21±3.88^fg^	85.23±.13^c^
CPCF17	13.68±3.86^def^	71.86±.62^fg^
CPCF18	8.21±3.87^fg^	64.53±1.25^ij^
CPCF19	8.21±3.87^fg^	79.19±.76^de^
CPCF20	19.16±3.88^bcd^	84.27±.08^c^
CPCF21	16.42±7.74^cde^	30.79±.47^n^
CPCF22	13.68±3.89^def^	40.53±.01^m^
CPCF23	8.21±3.89^fg^	10.35±.04°
CPCF24	8.21±0.35^fg^	84.34±7.67^c^
CPCF25	21.89±.00^bc^	54.65±.75^k^
CPCF26	10.94±.00^ef^	65.35±.88^ij^
CPCF27	13.68±0.73^def^	67.86±.04^hij^
CPCF28	13.68±0.45^def^	43.04±.86^m^
CPCF29	13.68±3.87^def^	70.04±.86^fgh^
CPCF30	5.47±.00^g^	90.09±.47^b^
CPCF31	13.68±3.85^def^	67.73±.23^hij^
CPCF32	8.20±3.89^fg^	70.40±.08^fgh^

Values are reported in mean ± SD. Means not sharing a common superscript letter across the column were significantly different (P<0.05). CPCF: Commercially Produced Complementary Foods

#### Oxalate content

The total oxalate content is also presented in Table 6. The total oxalate content of the commercial complementary foods varied from 5.47±0 to 30.10± 3.87 mg/100g. The highest content of total oxalate was found in a CPCF, which had a high amount of chickpea as its main ingredient. Standards show that a blended flour produced for the purpose of weaning food should have an oxalate content in the range of 40–50 mg/100 [23]. Based on this standard, none of the complementary foods surpassed the upper limit. The oxalate content of the present study showed lower results than those reported by Gemeda [41], but it is in accordance with Masazunu [27]. The low oxalate content in the formulated complementary flour is essential to inhibit oxalate binding to calcium to form calcium oxalate crystals, which may cause diseases such as oseteomalacia and rickets, particularly in infants and young children [41]. In addition to that, calcium oxalate crystals may have an important influence on the risk of formation kidney stone formation [45].

### Microbial count (yeast and mold count)

The yeast and mold count of the CPCFs is presented in Table 7. The yeast count was in the range of 0.00–3.65 log_10_ cfu/g, and the mold count ranged from 0.00–2.91 log_10_ cfu/g. Most of the mold and yeast count were not significantly different (P>0.05) from each other. The standard for yeast and mold in complementary foods had been reported to be less than 2.48 log_10_ cfu/g for ready-to-eat foods made for infants and 3 log_10_ cfu/g for foods that require cooking [23]. The permissible amounts of mold and yeast are between 25–250 cfu/g [46]. Based on these standards, only 1 out of the 32 products exceeded the limit for yeast, and none of the products had passed the limit set for molds. The result of the present study regarding mold and yeast is different from that of the reports conducted by Agbemafle et al. [31] and Mekuria et al. [26], where both mold and yeast were undetected. Another report showed that mold and yeast content of commercial wean mix has a log_10_ of 2.03 [47]. Achidi et al. [48] obtained 0.75 and 1.25 cfu/g10^2 for yeast and mold, respectively, which is lower than the current result.

**Table 7:** Microbial count of commercially produced complementary foods.

Treatment	Yeast (log_10_ cfu/g)	Mold (log_10_ cfu/g)
CPCF1	2.51±.00^ghi^	2.54±.01^cdefg^
CPCF2	2.1±.02^jk^	2.08±.01^fghijkl^
CPCF3	2.13±.03^j^	2.13±.02^efghijkl^
CPCF4	2.34±.00^i^	2.26±.03^defghij^
CPCF5	.00±.00°	.00±.00°
CPCF6	2.43±.03^hi^	2.49±.01^cdefgh^
CPCF7	2.40±.02^i^	2.56±.03^cdef^
CPCF8	2.49±.00^hi^	2.64±.00^abcd^
CPCF9	2.95±.08^bc^	2.45±.07^defgh^
CPCF10	2.71±.32^de^	2.35±.04^defghi^
CPCF11	1.85±.00^m^	2.55±.01^cdefg^
CPCF12	2.36±.03^i^	2.12±.03^fghijkl^
CPCF13	2.67±.06^def^	2.02±.02^ijklm^
CPCF14	.00±.00°	.00±.00°
CPCF15	1.74±.06^m^	1.8±.00^klmn^
CPCF16	1.82±.05^lm^	1.95±.07^ijklm^
CPCF17	3.65±.07^a^	2.66±.01^abcd^
CPCF18	2.93±.04^bc^	2.20±.14^defghijk^
CPCF19	2.82±.05^cd^	2.64±.01^abcd^
CPCF20	2.65±.07^efg^	2.14±.01^defghijk^
CPCF21	2.35±.01^i^	2.48±.02^defgh^
CPCF22	1.82±.05^lm^	1.91±.03^jklm^
CPCF23	1.98±.04^kl^	1.41±.06^n^
CPCF24	1.39±.13^n^	1.44±.04^n^
CPCF25	2.54 ±.09^fgh^	2.35±.06^defghi^
CPCF26	1.74±.06^m^	1.80±.03^klmn^
CPCF27	2.82±.05^cd^	2.91±.03^a^
CPCF28	1.93±.04^l^	1.61±.09^mn^
CPCF29	2.40±.02^hi^	2.58±.01^acde^
CPCF30	2.82±.04^bcd^	2.84±.13^abc^
CPCF31	1.93±.04^l^	1.71±.99^lmn^
CPCF32	2.43±.028^hi^	2.68±0.00^abcd^

Values are means ±SD; Means sharing a common superscript across the column aren’t significantly different. CPCF: Commercially Produced Complementary Foods

### Functional property of commercially produced complementary foods

#### Water absorbing capacity

The water-absorbing characteristics of the CPCFs ranged from 0.47±0.10 to 5.03±0.05 mL/g (Table 8). Besides, water absorption in baby porridge is assumed to be influenced by protein components and crude fiber content. Water absorption is one of the characteristics of protein hydration, namely the ability of proteins to hold water in a food system (low water absorption). Protein will cover starch particles so water absorption becomes inhibited [49] CPCF 5, 2, 6, and 7 were significantly different from the rest of the products (p<0.05). The water absorption capacity of the CPCFs were found to be very comparable to the value of complementary foods obtained by Usman et al. [50], which ranged from 2.01–3.81 ml/g. The WAC of a commercial complementary food was found to be 11.3% [51], which is higher than the current study, and this could be due to the higher variability of ingredients.

**Table 8:** Water absorbing character and Pasting property of the commercially produced complementary foods.

Samples	Water absorbing (mL/g)	Peak min Viscosity (RVU)	Break Down (RVU)	Final Viscosity (RVU)	Peak time (minutes)	Pasting temp (°C)
CPCF1	2.52±.00^j^	5215±21.21^c^	84±2.83^p^	671.5±3.54^s^	96.15±1.41^a^	71.06±1.48^f^
CPCF2	.47±.02^w^	2677.5±3.54^d^	367±4.24^i^	2574±5.66^d^	90.07±.03^b^	71.88±.6^e^
CPCF3	2.45±.01^jk^	58±2.83^q^	5±1.41^t^	58.5±2.12^z2^	95.55±1.20^a^	74±1.41^d^
CPCF4	3.07±.02^gh^	1310±14.14^h^	68±2.83^q^	2077±1.41^e^	95.7±.14^a^	58.28±1.17^p^
CPCF5	2.09±.02^n^	527±4.24^l^	233.5±16.26^k^	638.5±2.12^u^	95.1±.14^a^	61.53±1.17°
CPCF6	1.48±.01^r^	1287.5±3.54^h^	1028±8.49^d^	164±1.41^x^	95.88±1.17^a^	72.25±.49^de^
CPCF7	3.87±.02^d^	65.5±6.36^pq^	14±4.24^t^	63.5±2.12^z2^	89.25±1.63^bc^	73.5±.71^de^
CPCF8	5.03±.05^a^	183.5±3.54^n^	51±4.24^r^	35±4.24^z3^	90.73±.81^b^	55.45±.78^p^
CPCF9	.94±.00^t^	181±12.73^n^	51.5±3.54^r^	263.5±4.95^u^	90.75±1.06^b^	84.23±.67^a^
CPCF10	2.06±.02^no^	510.5±6.36^l^	83.5±2.12^p^	645.5±3.53^u^	90±1.06^b^	69.05±.78^gh^
CPCF11	2.53±.01^j^	1473±106.07^f^	1059.5±2.12^c^	765±4.24^q^	95.66±1.06^a^	70.83±.79^g^
CPCF12	3.04±.02^gh^	444±43.84^m^	75.5±4.95^pq^	697±4.24^r^	90±1.06^b^	77.82±.79^b^
CPCF13	3.1±.04^gh^	698.5±2.12^j^	150±5.66^n^	1140±1.41^l^	90.35±1.06^b^	67.09±1.05^ij^
CPCF14	1.82±.02^p^	565.5±17.68^l^	25.5±3.53^s^	866.5±3.54^n^	90.43±1.06^b^	76.29±.69^b^
CPCF15	2.01±.02^no^	187.5±17.68^n^	87.5±3.53^p^	134.5±3.54^y^	90.53±1.06^b^	80.18±1.17^b^
CPCF16	3.36±.04^f^	241.5±12.02^n^	76.5±4.95^pq^	209±1.4^w^	89.97±1.06^b^	55.45±.78^p^
CPCF17	3.16±.21^g^	65.5±6.36^pq^	12.5±2.12^t^	64±2.83^z2^	88.9±1.06^bc^	51±1.41^q^
CPCF18	.85±.01^ut^	158±12.73°	42.5±4.95^r^	105±7.07^z1^	90±1.06^b^	80.4±.42^b^
CPCF19	3.6±.02^e^	1971.5±9.19^e^	447.5±3.53^h^	2623±1.41^C^	90.6±1.06^b^	55.5±.71^p^
CPCF20	3.94±.01^d^	1289±86.27^h^	511.5±4.95^g^	1599.5±2.12^f^	95.06±1.06^a^	63.85±.49^n^
CPCF21	2.23±.02^m^	685±49.49^jk^	29±2.83^s^	1125±1.41^m^	90.35±1.06^b^	68.83±.25^hi^
CPCF22	2.37±.01^kl^	1390±11.31^g^	751.5±3.54^f^	1418±1.41^j^	95.3±1.06^a^	65.48±.32^klmn^
CPCF23	4.30±.02^c^	898.5±2.12^i^	188±2.83^m^	1277.5±2.12^k^	90.9±1.06^b^	65.45±.78^klmn^
CPCF24	2.65±.00^i^	945.5±3.54^i^	273.5±2.12^j^	1589±1.41^g^	96.45±1.06^a^	63.7±.28^n^
CPCF25	4.58±.01^b^	127±3.54^op^	49±2.83^r^	123.5±2.12^z^	90.4±1.06^b^	83.45±.49^a^
CPCF26	1.16±.21^s^	239±8.49^n^	76.5±4.95^pq^	211.5±4.95^w^	90.73±1.06^b^	55.3±.56^p^
CPCF27	3.02±.01^h^	1032±4.24^h^	213.5±2.12^l^	1479±1.4^h^	91.23±1.06^b^	64.5±1.34^mn^
CPCF28	2.57±.03^ij^	635±7.07^k^	147.5±3.54^n^	855±9.89°	90.83±1.06^b^	66.65±.35^jkl^
CPCF29	1.61±.01^q^	7930.5±31.82^b^	5508.5±.71^b^	3367.5±2.12^b^	87.55±1.06^c^	66.33±.53^klm^
CPCF30	.73±.01^v^	9732±5.66^a^	7088.5±16.26^a^	3476±5.66^a^	83.05±1.06^d^	65.05±.92^lmn^
CPCF31	1.94±.03°	1324.5±28.99^h^	944.5±3.53^e^	785±7.07^p^	95.73±1.06^a^	68.23±.81^hij^
CPCF32	2.28±.02^lm^	886±15.56^i^	203±2.83^l^	1432.5±3.53^i^	95.38±1.06^a^	69.05±.78^gh^

Values are means ±SD; Means sharing a common superscript across the column aren’t significantly different. RVU Rapid Viscosity Unit. CPCF: Commercially Produced Complementary Foods

### Viscosity of commercially produced complementary foods

The viscosity of various CPCFs is stated in Table 8. The peak viscosity (PV) ranged from 58±2.83–7930±31.82 RVU. A significant difference (P<0.05) was found on the majority of CPCFs. Only 31.25% of the samples fell under the permissible range of PV, which is 83–250 RVU, a value that determines easy swallowing for children and low and suitable infant feeding consistency [51]. Low PV implies that the weaning food forms a low-viscous paste rather than a thick gel on cooking and cooling. This means that the gruel has a high caloric density per unit volume rather than dietary bulk. High PV is an indication of high starch content and the ratio of amylase to amylopectin, as well as the resistance of the granules to swelling [52]. Chidi et al. [53] reported a peak viscosity of 45–235 RVU, and this indicated a lower result compared to the current study. The peak viscosity often correlates with the quality of the end product and also provides an indication of the viscosity. It is also advantageous, as the CPCFs would be watery and more solid could be added; this would amount to adding more nutrients and energy, which is better for growing children [54].

The breakdown viscosity of the samples ranged from 5 RVU to 7077 RVU (Table 8). The majority of the breakdown viscosity results showed a significant difference (p<0.05). The break-down viscosity of the current work was higher than that reported by Okorie et al. [55]. The lowest breakdown value (CPCF 3) indicated that the product is not thin when mixed with water to make a paste. Ikegwu et al. [52] reported that the lower the break-down viscosity, the higher the ability of the flour to withstand heating and shear stress during processing. The final viscosity (FV) of the products ranged from 35.00±4.24 to 3367.00±2.12 RVU. There was a significant difference among the CPCFs (P<0.05). FV is the most commonly used parameter to define the quality of a particular starch-based sample, as it indicates the material’s ability to form a viscous paste after cooking and cooling and the paste’s resistance to shear force during stirring. The highest FV value of 3367 RVU (CPCF 29) indicates the ability to form a firm viscoelastic paste or gel after cooking and cooling, owing to the association of starch molecules. The low FV value of 35 RVU (CPCF8) suggests that after cooking and cooling, the complementary diets form a low-viscous paste rather than a thick gel. [53].

The pasting temperature (PT) of the CPCFs (50–83.75°C) is presented in Table 8. There was no significant difference (p>0.05) among the majority of CPCFs. The high pasting temperature may be attributed to the higher starch or sugar content. Anosike et al., [56] reported pasting temperature of 90−92°C which was higher than the present study PT gives an indication of the gelatinization temperature during processing. It is the temperature at which the first detectable increase in viscosity is measured and is an index characterized by initial change due to the swelling of starch. The PT indicates the minimum temperature required for cooking and gelatinization Low gelatinization temperature implies shorter cooking time. It has been reported that the PT is related to water‐binding capacity). A higher PT implies higher gelatinization, higher water‐binding capacity and lower swelling property of starch due to a high degree of association between starch granules Ikegwu et al., [52].

The peak time indicates a cooking time for un-gelatinized starch; this could be the reason why it took a long time for the starch to attain peak viscosity [55]. According to Table 8, the peak time ranged from 6.49–7.63 minutes. The peak-time results also showed that almost all of the products had no significant difference from one another (P>0.05). Olagunju et al. [57] reported a peak time of 6.49–6.80, which showed similarity with most of the peak time results in this study. Thus, weaning food blends with a lower peak time will cook faster than those with a higher peak time.

### Association of different variables in commercially produced complementary foods

**Fig 1:**
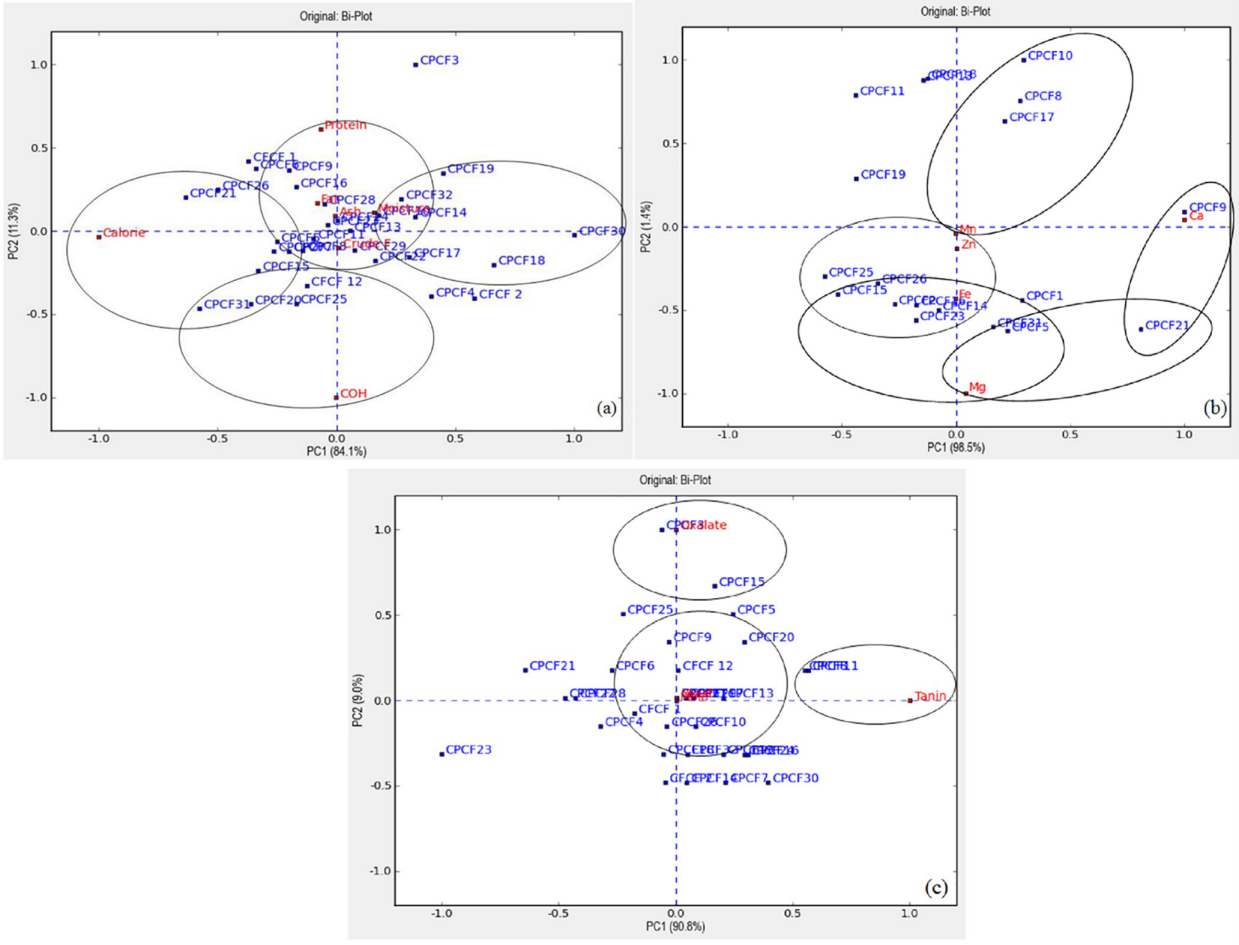
Principal component analysis predictive biplots of commercially produced complementary foods (CPCFs) over nutrition composition (a), commercially produced complementary foods and minerals (b), commercially produced complementary foods and ant-nutrients (c), and commercially produced complementary foods and aflatoxin B1 (d). The degree of proximity between variables and the narrower angle between diagonal lines indicated a strong association. CPCF = commercially produced complementary foods; Fe = iron; Zn = zinc; Ca = calcium; Mg = magnesium; Mn = manganese; AFB1 = aflatoxin B1; and EDI = estimate dietary intake of A.

An analysis was performed to look for the main data structures of the CPCFs and possible trends, as well as the degree of variations observed between variables (Fig 1). To carry out interpretations based on respective associations, PCA with predictive biplots was chosen. Fig 1A shows that PC1 explained 84.1% of the variation in the data set of the proximate content of the CPCFs, while PC2 explained 11.3%. The PCA biplots indicated that products like CPCF 13, 14, 32, 28, and 19 are in the right quadrants, and PCFs like 21, 26, 1, 9, and 16 are in the left quadrants. The products in the right-hand quadrants have associations with moisture, crude fiber, and ash. On the other hand, the products in the left quadrant have associations with calories, fat, and protein.

Fig 1B explains the association between the products and the mineral content. PC1 shows 98.5% variability, whereas PC2 shows 1.4%. The components show the mineral contents of the CPCFs, namely iron, zinc, calcium, magnesium and manganese. CPCF 1, 5 and 31 are rich in zinc, while products like CPCF26, 23 and 15 have a higher amount of Mn and relatively higher content of Fe compared to the other products. In contrast to that, products like CPCF 8, 10, 13, 11, 17 and 19 are found at a distance to go along with the minerals. The PCA plot for the minerals showed that all the minerals are in the right quadrant, and some products don’t show connotation with mineral composition.

Fig 1C explains the association between the CPCFs, the anti-nutrient, and the yeast and mold content of the products. PC1 has 90.8% variability, and PC2 shows 9%. Most of the products lie in the right quadrant, and products like CPCF25, 19, and 10 have a higher association with yeast presence, while mold content is related to CPCF4, 30, and 27.

## Conclusion and recommendation

Commercially produced complementary foods (CPCF) were investigated and computed in accordance with the standards. The study demonstrated that the CPCFs have lower-quality protein, energy, and fat. The mineral content of the samples indicated that all of the calcium and magnesium contents were below the standards specified. Despite the fact that some of the samples had optimal iron content, some products had iron and manganese values below the recommendation. Tannin and oxalate levels were both below the allowable limit. In addition to that, the yeast and mold counts showed that some products exceeded the permissible limit for each of the parameters. A PCA with predictive plots was used to plot the relationship between CPCFs and the parameters. The PCA biplot for proximate composition, mineral content, and microbial count showed the association between the products and the parameters supporting the current study. With few exceptions, CPCFs had low nutritional quality and safety. The study’s findings have several practical implications. First, because cereal-based CPCFs dominate the market, it is vital to include other ingredients, such as products made from animal sources and vegetables that are high in nutrients. Additionally, targeted fortification to improve the quality of CPCFs is crucial to ensuring that children are consuming the right amount of micronutrients. Furthermore, conducting regular market surveillance to evaluate the safety and quality of CPCFs to safeguard the consumer’s health is highly indispensable. The lack of phytate data, the small number of CPCFs used in the study, and the fact that the study did not assess the long-term effects of consuming CPCFs can be considered limitations of the study. Despite these limitations, the study’s findings provide important information about the nutritional quality and safety of CPCFs in Ethiopia. The findings can be used to improve the nutritional quality of CPCFs and ensure that children are getting the nutrients they need.

## Supporting information

S1 Table Proximate content of commercially produced complementary foods in (CPCFs) (g/100g). (DOCX)

S2 Table Mineral content of commercial complementary foods (CPCFs) in (mg/100g). (DOCX)

S3 Table Antinutrient content (CPCFs) (mg/100g), and yeast and mold content of commercially produced complementary foods (CPCFs) in log_10_ cfu/g. (DOCX)

S4 Table WAC and RVA of commercially produced complementary foods (CPCFs). (DOCX)

S5 Table List of vendors, manufacturer, lot number and purchase location of CPCF. (DOCX)

S1 Fig Calibration curve of zinc in the commercial complementary foods (CPCFs). (DOCX)

S2 Fig Calibration curve of calcium in the commercial complementary foods (CPCFs). (DOCX)

S3 Fig Calibration curve of manganese in the commercial complementary foods (CPCFs). (DOCX)

S4 Fig Calibration curve of magnesium in the commercial complementary foods (CPCFs). (DOCX)

S5 Fig Calibration curve of calcium in the commercial complementary foods (CPCFs). (DOCX)
