# Supplementary material for: Evaluation of the quality and safety of commercial complementary foods: Implications for nutrient adequacy and conformance with national and international standards
Source: PLoS One. 2024 Feb 21;19(2):e0294068. doi: 10.1371/journal.pone.0294068 (PMC10880965; doi:10.1371/journal.pone.0294068)
Supplement: S1 Table — (DOCX) [file pone.0294068.s001.docx]

S1 Appendix Table: Proximate content of commercially produced complementary foods in (CPCFs) (g/100g)

| **Products** | **Moisture** | **Ash** | **Fat** | **Protein** | **Crude Fiber** | **Carbohydrate** | **Calorie** |
| --- | --- | --- | --- | --- | --- | --- | --- |
| **CPCF1** | 3.78 | 2.33 | 6.38 | 12.52 | 2.28 | 72.71 | 398.28 |
| **CPCF1** | 3.18 | 2.33 | 5.87 | 13.1 | 2.40 | 73.11 | 397.73 |
| **CPCF2** | 11.80 | 0.80 | 0.50 | 0.70 | 3.40 | 86.20 | 352.10 |
| **CPCF2** | 10.20 | 0.80 | 0.75 | 1.13 | 3.49 | 87.12 | 359.75 |
| **CPCF3** | 7.00 | 4.67 | 4.12 | 17.78 | 2.50 | 64.43 | 365.96 |
| **CPCF3** | 7.39 | 4.67 | 4.25 | 18.54 | 2.48 | 63.16 | 365.04 |
| **CPCF4** | 7.00 | 3.33 | 0.75 | 2.45 | 13.10 | 86.46 | 362.42 |
| **CPCF4** | 7.30 | 3.00 | 1.25 | 2.89 | 13.30 | 85.57 | 365.03 |
| **CPCF5** | 4.59 | 2.00 | 2.50 | 7.27 | 3.10 | 83.64 | 386.12 |
| **CPCF5** | 5.19 | 1.67 | 5.49 | 8.53 | 3.20 | 79.13 | 400.01 |
| **CPCF6** | 5.39 | 1.67 | 6.00 | 13.05 | 2.50 | 73.90 | 401.78 |
| **CPCF6** | 6.37 | 2.00 | 5.12 | 12.35 | 2.56 | 74.15 | 392.13 |
| **CPCF7** | 3.20 | 1.00 | 1.50 | 10.42 | 1.63 | 83.88 | 390.70 |
| **CPCF7** | 3.60 | 1.00 | 1.75 | 9.89 | 1.64 | 83.76 | 390.35 |
| **CPCF8** | 0.60 | 1.60 | 1.75 | 9.51 | 2.84 | 83.70 | 388.57 |
| **CPCF8** | 1.00 | 1.40 | 1.38 | 10.50 | 2.76 | 82.97 | 386.25 |
| **CPCF9** | 7.60 | 2.67 | 6.48 | 10.30 | 3.30 | 72.96 | 391.31 |
| **CPCF9** | 7.40 | 2.67 | 6.11 | 10.20 | 3.35 | 73.62 | 390.28 |
| **CPCF10** | 5.00 | 2.00 | 0.87 | 11.56 | 3.3 | 80.57 | 376.37 |
| **CPCF10** | 6.40 | 2.33 | 1.00 | 10.95 | 3.34 | 79.32 | 370.07 |
| **CPCF11** | 4.80 | 1.67 | 2.87 | 8.84 | 2.6 | 81.81 | 388.51 |
| **CPCF11** | 6.20 | 1.33 | 2.75 | 8.41 | 2.65 | 81.31 | 383.62 |
| **CPCF12** | 4.00 | 1.33 | 2.13 | 6.22 | 3.10 | 86.32 | 389.29 |
| **CPCF12** | 4.00 | 2.00 | 1.88 | 5.69 | 3.12 | 86.43 | 385.38 |
| **CPCF13** | 5.60 | 2.00 | 1.25 | 9.89 | 2.4 | 81.26 | 375.85 |
| **CPCF13** | 4.60 | 2.00 | 1.63 | 10.34 | 2.43 | 81.43 | 381.73 |
| **CPCF14** | 7.00 | 3.67 | 0.88 | 9.81 | 3.3 | 78.65 | 361.71 |
| **CPCF14** | 7.00 | 1.20 | 0.75 | 10.14 | 3.36 | 80.91 | 370.95 |
| **CPCF15** | 0.03 | 2.67 | 1.37 | 9.02 | 5.10 | 86.91 | 396.10 |
| **CPCF15** | 0.03 | 2.67 | 1.37 | 11.30 | 5.3 | 84.63 | 396.09 |
| **CPCF16** | 5.00 | 1.00 | 5.00 | 11.38 | 3.10 | 74.52 | 388.60 |
| **CPCF16** | 4.00 | 1.00 | 4.37 | 10.87 | 3.13 | 76.63 | 389.35 |
| **CPCF17** | 2.60 | 2.67 | 0.63 | 7.09 | 3.90 | 83.12 | 366.46 |
| **CPCF17** | 2.60 | 2.67 | 0.87 | 6.74 | 3.93 | 83.19 | 367.59 |
| **CPCF18** | 13.2 | 0.80 | 1.38 | 1.23 | 1.04 | 83.40 | 350.88 |
| **CPCF18** | 13.4 | 0.80 | 2.13 | 1.35 | 1.03 | 82.32 | 353.83 |
| **CPCF19** | 9.60 | 0.40 | 0.50 | 12.78 | 2.1 | 76.72 | 362.50 |
| **CPCF19** | 10.2 | 0.80 | 0.75 | 13.40 | 2.12 | 74.85 | 359.75 |
| **CPCF20** | 0.05 | 3.00 | 1.13 | 7.62 | 2.3 | 88.21 | 393.42 |
| **CPCF20** | 0.05 | 1.33 | 1.50 | 7.18 | 2.36 | 89.94 | 401.97 |
| **CPCF21** | 5.40 | 2.67 | 7.87 | 8.64 | 2.4 | 75.42 | 407.11 |
| **CPCF21** | 4.40 | 2.67 | 8.50 | 8.61 | 2.42 | 75.83 | 414.23 |
| **CPCF22** | 5.00 | 2.00 | 0.87 | 7.27 | 2.4 | 84.86 | 376.37 |
| **CPCF22** | 6.40 | 2.33 | 1.38 | 6.74 | 2.41 | 83.15 | 371.94 |
| **CPCF23** | 6.20 | 1.00 | 2.75 | 8.93 | 2.6 | 81.12 | 384.95 |
| **CPCF23** | 6.80 | 1.67 | 3.13 | 9.37 | 2.61 | 79.04 | 381.76 |
| **CPCF24** | 5.60 | 1.00 | 1.63 | 10.68 | 2.2 | 81.09 | 381.73 |
| **CPCF24** | 5.80 | 1.33 | 1.88 | 11.30 | 2.21 | 79.70 | 380.84 |
| **CPCF25** | 2.40 | 2.67 | 2.63 | 3.41 | 3.10 | 88.90 | 392.86 |
| **CPCF25** | 3.20 | 3.33 | 2.37 | 4.50 | 3.14 | 86.59 | 385.74 |
| **CPCF26** | 5.30 | 2.33 | 6.00 | 10.95 | 3.15 | 75.42 | 399.47 |
| **CPCF26** | 4.30 | 2.00 | 6.87 | 10.86 | 3.16 | 75.97 | 409.17 |
| **CPCF27** | 2.80 | 2.00 | 2.50 | 9.37 | 2.30 | 83.33 | 393.30 |
| **CPCF27** | 4.00 | 1.33 | 3.00 | 8.67 | 2.36 | 83.00 | 393.67 |
| **CPCF28** | 6.40 | 2.00 | 2.75 | 10.86 | 2.60 | 77.99 | 380.15 |
| **CPCF28** | 4.60 | 2.33 | 3.00 | 11.56 | 2.61 | 78.51 | 387.27 |
| **CPCF29** | 6.80 | 1.00 | 1.25 | 9.02 | 3.10 | 81.93 | 375.05 |
| **CPCF29** | 5.10 | 1.00 | 1.13 | 8.32 | 3.30 | 84.46 | 381.23 |
| **CPCF30** | 17.2 | 0.40 | 1.37 | 0.96 | 1.10 | 80.06 | 336.47 |
| **CPCF30** | 17.4 | 0.80 | 2.13 | 1.14 | 1.30 | 78.54 | 337.83 |
| **CPCF31** | 4.40 | 1.33 | 6.12 | 1.14 | 3.10 | 87.00 | 407.69 |
| **CPCF31** | 4.60 | 1.33 | 6.63 | 1.05 | 3.16 | 86.39 | 409.39 |
| **CPCF32** | 7.00 | 2.33 | 1.25 | 11.38 | 2.70 | 78.03 | 368.92 |
| **CPCF32** | 7.00 | 2.00 | 1.00 | 11.73 | 2.76 | 78.27 | 369.00 |

CPCF: Commercially produced complementary food.
