# Supplementary material for: Evaluation of the quality and safety of commercial complementary foods: Implications for nutrient adequacy and conformance with national and international standards
Source: PLoS One. 2024 Feb 21;19(2):e0294068. doi: 10.1371/journal.pone.0294068 (PMC10880965; doi:10.1371/journal.pone.0294068)
Supplement: S2 Table — (DOCX) [file pone.0294068.s002.docx]

S2 Appendix Table: Mineral content of commercial complementary foods (CPCFs) in (mg/100g).

| **Products** | **Fe** | **Zn** | **Ca** | **Mg** | **Mn** | **EDIFe** | **EDIZn** | **EDICa** | **EDIMg** | **EDIMn** |
| --- | --- | --- | --- | --- | --- | --- | --- | --- | --- | --- |
| CPCF15 | 14.35 | 6.95 | 41.1 | 16.9 | 1.3 | 42.93 | 22.1 | 850.43 | 80.481 | 5.2 |
| CPCF15 | 13.9 | 6.6 | 44.2 | 16.8 | 1.3 | 41.81 | 21.2 | 828.73 | 80.232 | 5.2 |
| CPCF25 | 14.45 | 5.15 | 30 | 14.5 | 1.3 | 42.93 | 17.6 | 739.47 | 74.505 | 5.2 |
| CPCF25 | 14.25 | 4.9 | 31.1 | 14.4 | 1.25 | 42.68 | 17.0 | 723.32 | 74.256 | 5.0 |
| CPCF26 | 11.95 | 6.85 | 75.1 | 16.9 | 1.55 | 36.96 | 21.9 | 844.23 | 80.481 | 5.8 |
| CPCF26 | 16.55 | 6.65 | 84.1 | 17.3 | 1.25 | 48.49 | 21.4 | 831.83 | 81.477 | 5.0 |
| CPCF5 | 12.35 | 3.6 | 197.7 | 28.8 | 1.55 | 37.95 | 13.8 | 642.72 | 110.112 | 5.8 |
| CPCF5 | 12.75 | 4.1 | 202 | 28.4 | 1.55 | 38.95 | 15.8 | 692.94 | 109.116 | 5.8 |
| CPCF1 | 16.4 | 3.9 | 202 | 24.5 | 2.8 | 48.04 | 14.5 | 661.32 | 99.405 | 8.9 |
| CPCF1 | 13.55 | 3 | 225.2 | 24.8 | 3.2 | 40.94 | 12.3 | 605.52 | 100.152 | 9.9 |
| CPCF8 | 5.9 | 2.1 | 213.3 | 6.8 | 2.7 | 21.89 | 10.0 | 549.72 | 55.332 | 8.6 |
| CPCF8 | 3.4 | 2.1 | 211.5 | 6.5 | 0.85 | 15.67 | 10.0 | 549.72 | 54.585 | 4.0 |
| CPCF10 | 2.25 | 1.8 | 213.1 | 1.18 | 0.085 | 12.80 | 9.3 | 531.12 | 41.3382 | 2.1 |
| CPCF10 | 1.85 | 1.3 | 218.6 | 5.7 | 0.8 | 11.81 | 8.0 | 500.12 | 52.593 | 3.9 |
| CPCF9 | 8.1 | 7.75 | 356.8 | 23 | 0.065 | 27.37 | 24.1 | 900.03 | 95.67 | 2.1 |
| CPCF9 | 10.95 | 7.7 | 372.1 | 22.6 | 1.25 | 34.47 | 24.0 | 896.93 | 94.674 | 5.0 |
| CPCF11 | 1.45 | 0.7 | 51.4 | 1.2 | 1.95 | 10.81 | 6.5 | 462.92 | 41.388 | 6.8 |
| CPCF11 | 1.9 | 0.65 | 68.4 | 1.2 | 0.1 | 11.93 | 6.4 | 459.82 | 41.388 | 2.2 |
| CPCF14 | 10.45 | 4.25 | 136 | 24 | 0.1 | 33.22 | 15.4 | 683.02 | 98.16 | 2.2 |
| CPCF14 | 12.95 | 3.8 | 136.3 | 23.9 | 2.1 | 39.45 | 14.3 | 655.12 | 97.911 | 7.1 |
| CPCF16 | 7.6 | 3.8 | 115.7 | 23.7 | 2.1 | 26.12 | 14.3 | 655.12 | 97.413 | 7.1 |
| CPCF16 | 10.5 | 4.5 | 114.8 | 23.5 | 1.95 | 33.35 | 16.0 | 698.52 | 96.915 | 6.8 |
| CPCF17 | 1 | 0.5 | 197.7 | 9.6 | 2.4 | 9.69 | 6.0 | 450.52 | 62.304 | 7.9 |
| CPCF17 | 3.55 | 0.39 | 198.2 | 9.7 | 0.25 | 16.04 | 5.8 | 443.70 | 62.553 | 2.5 |
| CPCF31 | 11.95 | 0.72 | 187.9 | 26.9 | 0.8 | 36.96 | 6.6 | 464.16 | 105.381 | 3.9 |
| CPCF31 | 18.25 | 0.92 | 185.2 | 26.9 | 0.8 | 52.64 | 7.1 | 476.56 | 105.381 | 3.9 |
| CPCF2 | 10.25 | 1.04 | 95 | 22 | 2.4 | 32.72 | 7.4 | 484.00 | 93.18 | 7.9 |
| CPCF2 | 9.95 | 5 | 95.5 | 22.6 | 3.35 | 31.98 | 17.3 | 729.53 | 94.674 | 10.3 |
| CPCF9 | 7.4 | 3.7 | 60.4 | 8.7 | 1.8 | 25.63 | 14.0 | 648.92 | 60.063 | 6.4 |
| CPCF9 | 3.6 | 1.8 | 59.8 | 8.7 | 1.8 | 16.16 | 9.3 | 531.12 | 60.063 | 6.4 |
| CPCF21 | 10.65 | 5.7 | 323.7 | 34.1 | 1.3 | 33.72 | 19.0 | 772.93 | 123.309 | 5.2 |
| CPCF21 | 10.75 | 4.5 | 323.1 | 34.3 | 0.7 | 33.97 | 16.0 | 698.52 | 123.807 | 3.7 |
| CPCF23 | 8 | 4.7 | 114.5 | 25.5 | 2.65 | 27.12 | 16.5 | 710.92 | 101.895 | 8.5 |
| CPCF23 | 8.95 | 4.6 | 115.3 | 25.4 | 1.8 | 29.49 | 16.3 | 704.72 | 101.646 | 6.4 |
| CPCF18 | 5.65 | 0.35 | 127.4 | 0 | 0.3 | 21.27 | 5.7 | 441.22 | 0 | 2.7 |
| CPCF18 | 7.35 | 1.4 | 126.1 | 0 | 0.3 | 25.50 | 8.3 | 506.32 | 0 | 2.7 |
| CPCF13 | 1.35 | 1.900 | 123.5 | 2 | 0.2 | 10.56 | 9.5 | 537.32 | 43.38 | 2.4 |
| CPCF13 | 1.4 | 1.9 | 121.7 | 2.2 | 0.2 | 10.69 | 9.5 | 537.32 | 43.878 | 0.0 |

EDI: Estimated daily intake, Fe: Iron, Zn: Zinc, Ca: Calcium, Mg: Magnesium, Mn: Manganese,
