# Supplementary material for: Evaluation of the quality and safety of commercial complementary foods: Implications for nutrient adequacy and conformance with national and international standards
Source: PLoS One. 2024 Feb 21;19(2):e0294068. doi: 10.1371/journal.pone.0294068 (PMC10880965; doi:10.1371/journal.pone.0294068)
Supplement: S3 Table — (DOCX) [file pone.0294068.s003.docx]

S3 Appendix Table: Antinutrient content (CPCFs) (mg/100g), and Yeast and mold content of commercially produced complementary foods (CPCFs) in log10 cfu/g

| **Products** | **Oxalate** | **Tanin** | **Yeast** | **Mold** |
| --- | --- | --- | --- | --- |
| **CPCF1** | 10.94 | 58.7 | 2.51 | 2.53 |
| **CPCF1** | 13.44 | 56.2 | 2.51 | 2.55 |
| **CPCF2** | 5.47 | 63.4 | 2.08 | 2.07 |
| **CPCF2** | 5.47 | 66.7 | 2.11 | 2.08 |
| **CPCF3** | 27.36 | 64.2 | 2.15 | 2.12 |
| **CPCF3** | 32.83 | 64.1 | 2.11 | 2.14 |
| **CPCF4** | 10.94 | 48.3 | 2.34 | 2.28 |
| **CPCF4** | 10.94 | 50.1 | 2.34 | 2.24 |
| **CPCF5** | 21.89 | 81.2 | 2.36 | 0 |
| **CPCF5** | 21.89 | 81.9 | 2.36 | 0 |
| **CPCF6** | 16.42 | 50.1 | 2.41 | 2.5 |
| **CPCF6** | 16.42 | 53.9 | 2.45 | 2.48 |
| **CPCF7** | 5.47 | 78.3 | 2.38 | 2.54 |
| **CPCF7** | 5.47 | 81.2 | 2.41 | 2.58 |
| **CPCF8** | 16.42 | 98.5 | 2.49 | 2.64 |
| **CPCF8** | 16.42 | 99.6 | 2.49 | 2.64 |
| **CPCF9** | 21.89 | 65.1 | 3 | 2.4 |
| **CPCF9** | 16.42 | 66.7 | 2.89 | 2.5 |
| **CPCF10** | 10.94 | 72.3 | 2.93 | 2.37 |
| **CPCF10** | 10.94 | 72.5 | 2.48 | 2.33 |
| **CPCF11** | 16.42 | 99.6 | 1.85 | 2.54 |
| **CPCF11** | 16.42 | 100.3 | 1.85 | 2.56 |
| **CPCF12** | 16.42 | 67.3 | 2.34 | 2.11 |
| **CPCF12** | 16.42 | 68.96 | 2.38 | 2.14 |
| **CPCF13** | 10.94 | 78.23 | 2.63 | 2 |
| **CPCF13** | 16.42 | 80.13 | 2.71 | 2.03 |
| **CPCF14** | 5.47 | 70.1 | 0 | 0 |
| **CPCF14** | 5.47 | 70.4 | 0 | 0 |
| **CPCF15** | 21.89 | 76.51 | 1.7 | 1.8 |
| **CPCF15** | 27.36 | 77.58 | 1.78 | 1.8 |
| **CPCF16** | 5.47 | 85.14 | 1.78 | 1.9 |
| **CPCF16** | 10.94 | 85.32 | 1.85 | 2 |
| **CPCF17** | 10.94 | 71.42 | 3.7 | 2.65 |
| **CPCF17** | 16.42 | 72.3 | 3.6 | 2.67 |
| **CPCF18** | 5.47 | 63.64 | 2.9 | 2.28 |
| **CPCF18** | 10.94 | 65.41 | 2.95 | 2.12 |
| **CPCF19** | 5.47 | 78.65 | 2.78 | 2.65 |
| **CPCF19** | 10.94 | 79.72 | 2.85 | 2.63 |
| **CPCF20** | 16.42 | 84.21 | 2.6 | 2.15 |
| **CPCF20** | 21.89 | 84.32 | 2.7 | 2.13 |
| **CPCF21** | 10.94 | 30.45 | 2.34 | 2.46 |
| **CPCF21** | 21.89 | 31.12 | 2.36 | 2.49 |
| **CPCF22** | 10.94 | 40.52 | 1.78 | 1.93 |
| **CPCF22** | 16.42 | 40.54 | 1.85 | 1.9 |
| **CPCF23** | 5.47 | 10.32 | 1.95 | 1.41 |
| **CPCF23** | 10.94 | 10.38 | 2 | 1.47 |
| **CPCF24** | 10.94 | 89.76 | 1.48 | 1.41 |
| **CPCF24** | 5.47 | 78.91 | 1.3 | 1.46 |
| **CPCF25** | 21.89 | 54.12 | 2.6 | 2.31 |
| **CPCF25** | 21.89 | 55.18 | 2.48 | 2.39 |
| **CPCF26** | 10.94 | 65.98 | 1.7 | 1.78 |
| **CPCF26** | 10.94 | 64.73 | 1.78 | 1.82 |
| **CPCF27** | 10.94 | 67.89 | 2.85 | 2.89 |
| **CPCF27** | 16.42 | 67.83 | 2.78 | 2.93 |
| **CPCF28** | 16.42 | 43.64 | 1.95 | 1.67 |
| **CPCF28** | 10.94 | 42.43 | 1.9 | 1.56 |
| **CPCF29** | 10.94 | 70.65 | 2.38 | 2.57 |
| **CPCF29** | 16.42 | 69.43 | 2.41 | 2.59 |
| **CPCF30** | 5.47 | 90.42 | 2.79 | 2.93 |
| **CPCF30** | 5.47 | 89.75 | 2.85 | 2.75 |
| **CPCF31** | 16.42 | 67.89 | 1.95 | 0.71 |
| **CPCF31** | 10.94 | 67.56 | 1.9 | 2.71 |
| **CPCF32** | 10.94 | 70.34 | 2.41 | 2.68 |
| **CPCF32** | 5.47 | 70.45 | 2.45 | 2.68 |
