# Supplementary material for: Evaluation of the quality and safety of commercial complementary foods: Implications for nutrient adequacy and conformance with national and international standards
Source: PLoS One. 2024 Feb 21;19(2):e0294068. doi: 10.1371/journal.pone.0294068 (PMC10880965; doi:10.1371/journal.pone.0294068)
Supplement: S4 Table — (DOCX) [file pone.0294068.s004.docx]

S4 Appendix Table: WAC and RVA of commercially produced complementary foods (CPCFs)

| **Products** | **WAC (**ml/g) | **Peak Viscosity**  **(RVU)** | **Breakdown**  **(RVU)** | **F.viscosity (RVU)** | **Peaktime**  **(Min)** | **Pasting (T^0)^** |
| --- | --- | --- | --- | --- | --- | --- |
| **CPCF1** | 2.52 | 5230 | 82 | 669 | 95.15 | 70.01 |
| **CPCF1** | 2.52 | 5200 | 86 | 674 | 97.15 | 72.1 |
| **CPCF2** | 0.48 | 2680 | 364 | 2570 | 90.05 | 71.45 |
| **CPCF2** | 0.45 | 2675 | 370 | 2578 | 90.09 | 72.3 |
| **CPCF3** | 2.46 | 56 | 4 | 57 | 94.7 | 73 |
| **CPCF3** | 2.44 | 60 | 6 | 60 | 96.4 | 75 |
| **CPCF4** | 3.08 | 1300 | 66 | 2076 | 95.1 | 57.45 |
| **CPCF4** | 3.05 | 1320 | 70 | 2078 | 96.3 | 59.1 |
| **CPCF5** | 2.07 | 524 | 222 | 637 | 95 | 60.75 |
| **CPCF5** | 2.10 | 530 | 245 | 640 | 95.2 | 62.3 |
| **CPCF6** | 1.48 | 1285 | 1022 | 163 | 95.05 | 72.6 |
| **CPCF6** | 1.47 | 1290 | 1034 | 165 | 96.7 | 71.9 |
| **CPCF7** | 3.85 | 61 | 11 | 62 | 88.1 | 73 |
| **CPCF7** | 3.88 | 70 | 17 | 65 | 90.4 | 74 |
| **CPCF8** | 4.99 | 181 | 48 | 32 | 90.15 | 54.9 |
| **CPCF8** | 5.06 | 186 | 54 | 38 | 91.3 | 56 |
| **CPCF9** | 0.94 | 172 | 49 | 260 | 90 | 83.75 |
| **CPCF9** | 0.94 | 190 | 54 | 267 | 91.5 | 84.7 |
| **CPCF10** | 2.04 | 506 | 82 | 643 | 90 | 68.5 |
| **CPCF10** | 2.07 | 515 | 85 | 648 | 90 | 69.6 |
| **CPCF11** | 2.52 | 1398 | 1058 | 762 | 95.15 | 70.05 |
| **CPCF11** | 2.54 | 1548 | 1061 | 768 | 96.16 | 71.6 |
| **CPCF12** | 3.05 | 413 | 72 | 694 | 90 | 77.25 |
| **CPCF12** | 3.02 | 475 | 79 | 700 | 90 | 78.38 |
| **CPCF13** | 3.12 | 697 | 146 | 1139 | 90 | 66.35 |
| **CPCF13** | 3.07 | 700 | 154 | 1141 | 90.7 | 67.84 |
| **CPCF14** | 1.80 | 553 | 23 | 864 | 90.05 | 75.8 |
| **CPCF14** | 1.83 | 578 | 28 | 869 | 90.8 | 76.78 |
| **CPCF15** | 2.02 | 175 | 85 | 132 | 90.05 | 79.35 |
| **CPCF15** | 1.99 | 200 | 90 | 137 | 91 | 81 |
| **CPCF16** | 3.33 | 233 | 73 | 208 | 89.85 | 54.9 |
| **CPCF16** | 3.38 | 250 | 80 | 210 | 90.09 | 56 |
| **CPCF17** | 3.30 | 61 | 11 | 62 | 88.1 | 50 |
| **CPCF17** | 3.01 | 70 | 14 | 66 | 89.7 | 52 |
| **CPCF18** | 0.86 | 149 | 39 | 100 | 90 | 80.1 |
| **CPCF18** | 0.84 | 167 | 46 | 110 | 90 | 80.7 |
| **CPCF19** | 3.58 | 1965 | 445 | 2622 | 90 | 55 |
| **CPCF19** | 3.61 | 1978 | 450 | 2624 | 91.2 | 56 |
| **CPCF20** | 3.95 | 1228 | 508 | 1598 | 95.05 | 63.5 |
| **CPCF20** | 3.93 | 1350 | 515 | 1601 | 95.07 | 64.2 |
| **CPCF21** | 2.24 | 650 | 27 | 1124 | 90 | 68.65 |
| **CPCF21** | 2.21 | 720 | 31 | 1126 | 90.7 | 69 |
| **CPCF22** | 2.37 | 1382 | 749 | 1417 | 95 | 65.25 |
| **CPCF22** | 2.36 | 1398 | 754 | 1419 | 95.6 | 65.7 |
| **CPCF23** | 4.28 | 897 | 186 | 1276 | 90 | 64.9 |
| **CPCF23** | 4.31 | 900 | 190 | 1279 | 91.8 | 66 |
| **CPCF24** | 2.65 | 943 | 272 | 1588 | 95.1 | 63.5 |
| **CPCF24** | 2.65 | 948 | 275 | 1590 | 97.8 | 63.9 |
| **CPCF25** | 4.58 | 125 | 47 | 122 | 89.9 | 83.1 |
| **CPCF25** | 4.57 | 130 | 51 | 125 | 90.9 | 83.8 |
| **CPCF26** | 1.30 | 233 | 73 | 208 | 89.85 | 54.9 |
| **CPCF26** | 1.01 | 245 | 80 | 215 | 91.6 | 55.7 |
| **CPCF27** | 3.03 | 1029 | 212 | 1478 | 90.05 | 63.55 |
| **CPCF27** | 3.01 | 1035 | 215 | 1480 | 92.4 | 65.45 |
| **CPCF28** | 2.59 | 630 | 145 | 848 | 90.05 | 66.4 |
| **CPCF28** | 2.55 | 640 | 150 | 862 | 91.6 | 66.9 |
| **CPCF29** | 1.60 | 7908 | 5508 | 3366 | 86.4 | 65.95 |
| **CPCF29** | 1.62 | 7953 | 5509 | 3369 | 88.7 | 66.7 |
| **CPCF30** | 0.74 | 9728 | 7077 | 3472 | 83.05 | 64.4 |
| **CPCF30** | 0.72 | 9736 | 7100 | 3480 | 83.05 | 65.7 |
| **CPCF31** | 1.92 | 1304 | 942 | 780 | 95.15 | 67.65 |
| **CPCF31** | 1.96 | 1345 | 947 | 790 | 96.3 | 68.8 |
| **CPCF32** | 2.26 | 875 | 201 | 1430 | 94.85 | 68.5 |
| **CPCF32** | 2.29 | 897 | 205 | 1435 | 95.9 | 69.6 |

WAC: Water absorption content, RVU: Rapid visco analyzer unit,
