# Supplementary material for: Evaluation of the quality and safety of commercial complementary foods: Implications for nutrient adequacy and conformance with national and international standards
Source: PLoS One. 2024 Feb 21;19(2):e0294068. doi: 10.1371/journal.pone.0294068 (PMC10880965; doi:10.1371/journal.pone.0294068)
Supplement: S5 Table — (DOCX) [file pone.0294068.s005.docx]

S5 Appendix Table: List of vendors, manufacturer, lot number and purchase location of CPCF

| Treatment | Vendors | Manufacturer | Lot Number | Purchase Location |
| --- | --- | --- | --- | --- |
| CPCF1 | ^Shola Gebeya^ | * | 37 | ^Shola^ |
| CPCF2 | ^Abadir Supermarket^ | * | 259 | ^4 kilo^ |
| CPCF3 | ^Shola Gebeya^ | * | NA | ^Shola^ |
| CPCF4 | ^Abadir Supermarket^ | * | NA | ^4 kilo^ |
| CPCF5 | ^Abadir Supermarket^ | * | 18 | ^4 kilo^ |
| CPCF6 | ^Abadir Supermarket^ | * | NA | ^4 kilo^ |
| CPCF7 | ^Shewa Supermarket^ | * | 370920 | ^St. Gebriel^ |
| CPCF8 | ^Shewa Supermarket^ | * | 360920 | ^St. Gebriel^ |
| CPCF9 | ^Shewa Supermarket^ | * | 0321001 | ^St. Gebriel^ |
| CPCF10 | ^Shola Gebeya^ | * | NA | ^Shola^ |
| CPCF11 | ^Shola Gebeya^ | * | NA | ^Shola^ |
| CPCF12 | ^Shola Gebeya^ | * | NA | ^Shola^ |
| CPCF13 | ^Shola Gebeya^ | * | NA | ^Shola^ |
| CPCF14 | ^Shewa Supermarket^ | * | NA | ^Megegnaga^ |
| CPCF15 | ^Abadir Supermarket^ | * | 1020098 | ^4 kilo^ |
| CPCF16 | ^Shewa Supermarket^ | * | 012021 | ^St. Gebriel^ |
| CPCF17 | ^Shewa Supermarket^ | * | 502021 | ^St. Gebriel^ |
| CPCF18 | ^Shola Gebeya^ | * | NA | ^Shola^ |
| CPCF19 | ^Shola Gebeya^ | * | NA | ^Shola^ |
| CPCF20 | ^Shola Gebeya^ | * | NA | ^Shola^ |
| CPCF21 | ^Shola Gebeya^ | * | NA | ^Shola^ |
| CPCF22 | ^Shola Gebeya^ | * | NA | ^Shola^ |
| CPCF23 | ^Shola Gebeya^ | * | NA | ^Shola^ |
| CPCF24 | ^Shola Gebeya^ | * | NA | ^Shola^ |
| CPCF25 | ^Shewa Supermarket^ | * | 1110098 | ^St. Gebriel^ |
| CPCF26 | ^Shewa Supermarket^ | * | 0820032 | ^St. Gebriel^ |
| CPCF27 | ^Shola Gebeya^ | * | NA | ^Shola^ |
| CPCF28 | ^Shola Gebeya^ | * | NA | ^Shola^ |
| CPCF29 | ^Shola Gebeya^ | * | NA | ^Shola^ |
| CPCF30 | ^Shola Gebeya^ | * | NA | ^Shola^ |
| CPCF31 | ^Shewa Supermarket^ | * | NA | ^Megegnaga^ |
| CPCF32 | ^Abadir Supermarket^ | * | 03068 | ^4kilo^ |

* The manufacturers are not disclosed due to confidential reason.

N/A: Not available
