# Supplementary figures and images for: Evaluation of the quality and safety of commercial complementary foods: Implications for nutrient adequacy and conformance with national and international standards
Source: PLoS One. 2024 Feb 21;19(2):e0294068. doi: 10.1371/journal.pone.0294068 (PMC10880965; doi:10.1371/journal.pone.0294068)

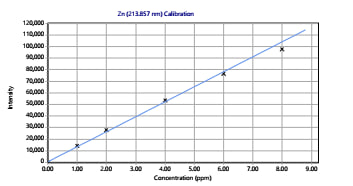


S1 Appendix Figure: Calibration curve of zinc in the commercial complementary foods (CPCFs)

Supplement: S1 Fig — (DOCX) [file pone.0294068.s006.docx]

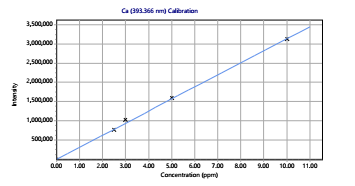


S2 Appendix Figure: Calibration curve of calcium in the commercial complementary foods (CPCFs)

Supplement: S2 Fig — (DOCX) [file pone.0294068.s007.docx]

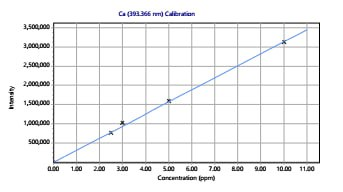


S5. Appendix Figure: Calibration curve of calcium in the commercial complementary foods (CPCFs)

Supplement: S5 Fig — (DOCX) [file pone.0294068.s010.docx]
