# Supplementary material for: Evaluation of the quality and safety of commercial complementary foods: Implications for nutrient adequacy and conformance with national and international standards
Source: PLoS One. 2024 Feb 21;19(2):e0294068. doi: 10.1371/journal.pone.0294068 (PMC10880965; doi:10.1371/journal.pone.0294068)
Supplement: S4 Fig — (DOCX) [file pone.0294068.s009.docx]

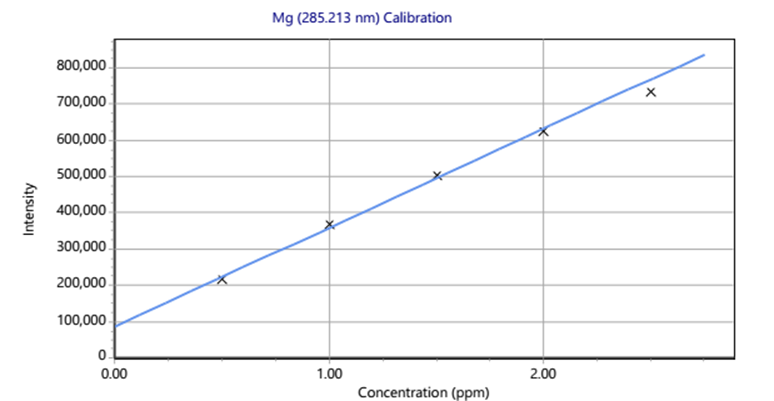


S4. Appendix Figure: Calibration curve of magnesium in the commercial complementary foods (CPCFs)
